# Fascioliasis in Kenya: a review

**DOI:** 10.3389/fvets.2026.1998426

**Published:** 2026-10-26

**Authors:** Victor O. Magero, Gabriel O. Dida

**Affiliations:** 1 School of Life Sciences, University of Nottingham, Nottingham, United Kingdom; 2 Institute of Tropical Medicine, Mount Kenya University, Thika, Kenya; 3 Department of Health Systems Management & Public Health, The Technical University of Kenya, Nairobi, Kenya; 4 Department of Public Health & Community Development, Maseno University, Maseno, Kenya

**Keywords:** *Fasciola* spp., Fascioliasis, *Galba* spp., Kenya, One Health, *Radix natalensis*

## Abstract

Fascioliasis is a disease of major public health, veterinary health and socio-economic importance in Kenya. The disease is classified as a Neglected Tropical Disease by the World Health Organisation with *Fasciola hepatica* and *Fasciola gigantica* being the leading causative agents of fascioliasis in the world. The primary definitive hosts of *Fasciola* parasites are domestic ruminants with humans being accidentally infected and mud snails of genera *Radix* spp. and *Galba* spp. serving as the intermediate hosts. However, information regarding fascioliasis in Kenya is sparse and scattered. A structured narrative review of fascioliasis in Kenya was conducted with an aim of addressing socio-economic factors associated with fascioliasis, the economic impact of fascioliasis, snail intermediate hosts that support the transmission of the disease, high risk areas/high endemic areas, wildlife ruminant hosts, control efforts used in curtailing transmission of fascioliasis, diagnosis and finally treatment of fascioliasis in Kenya. Relevant literature was identified through searches of PubMed/MEDLINE (*n* = 5,003), Web of Science (*n* = 9,014), Scopus (*n* = 6,530) and Google scholar/WHO/other sources (*n* = 7,510) using specific search terms and Boolean operators. Quality was assessed using the Joanna Briggs Institute (JBI) critical appraisal tools matched to each study design, with SANRA guidelines being used to improve transparency and reproducibility. Studies were considered eligible when they reported findings directly relevant to fascioliasis in Kenya with particular emphasis being placed on primary studies that provided quantitative evidence on fascioliasis in Kenya. The final review included 72 studies. It was ascertained that poverty and rural communities are the two major socio-economic factors associated with fascioliasis in Kenya with the disease being shown to disproportionately affect impoverished and rural communities that have limited access to clean water and healthcare services. Water projects such as dams and irrigation schemes were also found to influence the occurrence and prevalence of fascioliasis in Kenya. *Fasciola gigantica* was found to be the most predominant *Fasciola* parasite that causes fascioliasis in Kenya with *Fasciola hepatica* being found to be less prevalent. Important snail intermediate hosts that were found as being vital for the transmission of fascioliasis in Kenya were *Radix natalensis* and *Galba* spp. snails. Financial losses instigated by liver condemnation due to *Fasciola* (liver fluke) infections was found to be one of the major negative economic impacts of fascioliasis in Kenya, followed by inflated agricultural operating costs, treatment costs, veterinary fees and increased labour costs spurred by the disease. Reduced productivity of livestock due to chronic fascioliasis also translates to economic losses. In regard to high risk areas, the former Western province was found to be the province with the highest risk of fascioliasis in Kenya. In terms of wild-ruminant hosts, elephants, African buffaloes and camels are the only ones to have been reported infected with fascioliasis. It was also ascertained that diagnosis of fascioliasis in Kenya primarily relies on coprological sedimentation techniques to detect *Fasciola* eggs in faeces, with less use of immunological and molecular techniques. Control efforts used to interrupt transmission of fascioliasis in Kenya involves improved animal husbandry practices such as feeding livestock clean pasture, home watering of livestock, routine deworming and snail control. Finally, it was ascertained that treatment of fascioliasis in Kenya primarily utilises Triclabendazole, the drug recommended by the World Health Organisation with plant anthelmintics slowly being adopted as an alternative because of costs associated with Triclabendazole, potential development of anthelmintic resistance and availability of easy to use plant resources. Based on the results herein, an integrated “One-Health” approach in the fight against fascioliasis in Kenya is highly recommended.

## 1 Introduction

### 1.1 Background information

Fascioliasis is a zoonotic, parasitic, infectious disease caused by liver flukes that infect domestic ruminants, wild ruminants and humans with the disease linked to serious economic and health challenges (1–3). Fascioliasis is classified by the World health Organization as 1 of the 4 major Food borne trematodiasis, with the other 3 being Paragonimiasis, Clonorchiasis and Opisthorchiasis (4, 5). *Fasciola hepatica* and *F. gigantica* are the leading causative agents of fascioliasis in the world (6) with *F. hepatica* having a worldwide distribution but predominating temperate zones whereas *F. gigantica* being primarily found in tropical regions (7). Interestingly, in subtropical regions, *F. hepatica* and *F. gigantica* overlap (8, 9). The primary definitive hosts of *Fasciola* parasites are domestic ruminants (10), with humans being accidentally infected (104). While in the definitive hosts, *Fasciola* parasites migrate to the bile duct and gall bladder of the hosts, severely damaging these organs (11). Humans and animals get infected with fascioliasis by ingesting water, food and watercress or vegetation contaminated with metacercariae infective stages of *Fasciola* parasites (11). Prevalences of 30 – 90% *Fasciola* infection have been reported in ruminants from the African continent (12) and estimates suggest that over 600 animals globally at risk of *Fasciola* infection (13, 14, 105).

Mud snails, specifically of genera *Radix* spp. and *Galba* spp. which thrive in wet environments are utilised by *Fasciola* parasites, to complete their life cycle (Refs). *Radix* spp. (such as *Radix natalensis* or *Radix auricularia*) prefer more permanent aquatic habitats and act as the principal vectors for *F. gigantica* (the tropical liver fluke) whereas *Galba* (most notably *Galba truncatula*) thrive in amphibious, mud-laden, or temporary wetland environments and are the primary intermediate hosts for *F. hepatica* (15, 16) (Mahulu et al., 2019).

Human fascioliasis is a problem in areas where fascioliasis is present in livestock and where people in those localities eat aquatic or semi-aquatic plants and livestock or human faeces contaminate water bodies; with a direct connection to poor sanitation and inadequate food hygiene (17). Approximately 180 million people in 75 countries of the world are at risk of contracting fascioliasis, with estimates of over 2.4 million people currently infected (13, 17, 18). Human fascioliasis is characterised by distinct clinical symptoms divided into an acute (migratory) phase and a chronic (biliary) phase (8). The acute phase happens when juvenile liver flukes migrate through the intestinal wall and liver tissue and symptoms typically appear weeks after eating contaminated plants or water. Symptoms of acute fasciolisis include fever, abdominal pain, digestive issues, allergic reactions, general malaise, enlarged liver and high eosinophil count. The chronic phase on the other hand begins when adult flukes settle inside the bile ducts, months to years after the initial infection and the chronic symptoms include biliary colic, inflammation of the bile ducts, inflammation of the gallbladder or blockage of the gallbladder courtesy of gallstones (8, 19).

In Kenya, fascioliais is primarily caused by *F. gigantica*, which is common in tropical areas, and occasionally caused by *F. hepatica* in high-altitude zones (20, 21). Snail intermediate hosts involved in the transmission of *F. gigantica* and *F. hepatica* in Kenya are *Radix natalensis* and *Galba* spp., respectively (22, 23). Formal clinical human data on food-borne trematodiases in Kenya remains scarce and data on infection rates in livestock is limited (3, 24–26). In addition, there are a few recorded epidemiological data on bovine fascioliasis in Kenya, despite the disease being of great economic importance to livestock farming (25).

Information regarding fascioliasis in Kenya is sparse and scattered. For a proper integrated approach in the fight against fascioliasis in Kenya, as recommended by the World Health Organisation, there is a need for aggregated information that would potentially be used by public health officials and veterinarians in the fight against fascioliasis. This disease requires a “One Health” approach that coordinates public health officials and veterinarians to achieve successful control. This review intends to aggregate the current available information on fascioliasis in Kenya from scholarly articles and give the disease the attention it deserves.

## 2 Methodology

### 2.1 Search strategy

A structured narrative review of Fascioliasis in Kenya was conducted. The primary literature search focused on published peer-reviewed studies. The search did not include a specific period because scholarly articles on this topic are first and foremost scarce. Relevant literature was identified through searches of PubMed/MEDLINE, Web of Science and Scopus. Additional publications and technical documents were identified from Google Scholar, the World Health Organization (WHO) and relevant regulatory or public-health sources where appropriate. Reference lists of key reviews and primary studies were also examined to identify additional relevant publications.

Search terms were organised according to the major themes of the review and combined using Boolean operators (AND/OR). Core terms included “fascioliasis,” “Fascioliasis in Kenya”, “*Fasciola* spp.”, “*F. gigantica*”, “*F. hepatica*”, “Socio-economic factors”, “diagnosis”, “Coprological analysis”, “Sedimentation technique”, “Water projects”, “Irrigation schemes”, “Economic impact”, “High risk areas”, “Wildlife Ruminant hosts”, “prevention”, “preventive chemotherapy”, “mass drug administration”, “Triclabendazole”, “Plant anthelmintics”, “Synthetic molluscicides”, “Plant molluscicides”, “Control efforts”, “Snail control”, “molluscicides”, “Triclabendazole”,” anthelmintics” and “One-Health.” The search strategy was designed to provide broad coverage of fascioliasis in Kenya rather than to fulfil the requirements of a formal systematic review or meta-analysis. The literature identification and selection process was documented to improve transparency and reproducibility, consistent with recommended quality criteria for narrative reviews as per the SANRA (Scale for the Assessment of Narrative Review Articles) guidelines by Baethge et al. (27).

### 2.2 Eligibility and study selection

Studies were considered eligible when they reported findings directly relevant to fascioliasis in Kenya. Eligible publications included randomised and non-randomised clinical trials, diagnostic accuracy studies, epidemiological and surveillance studies, field intervention studies, molecular and genomic investigations, modelling studies, and relevant systematic or narrative reviews. Joanna Briggs Institute (JBI) critical appraisal tool was use to appraise the quality studies (102, 103), and matched to each study design, with SANRA guidelines being used to improve transparency and reproducibility (27).

Particular emphasis was placed on primary studies that provided quantitative evidence on intervention effectiveness, diagnostic performance, treatment efficacy, transmission dynamics or clinical outcomes. Recent systematic reviews and authoritative WHO documents were used to contextualise primary evidence and identify broader trends in fascioliasis control and elimination.

Studies were excluded if they were unrelated to fascioliasis in Kenya, lacked sufficient relevance to prevention, diagnosis, surveillance or treatment, duplicated previously reported findings without substantial new data, or were not available in English. Studies involving animal hosts or snail intermediate hosts were retained when they provided information relevant to zoonotic transmission, One Health, snail control, prevention or interruption of transmission.

Titles and abstracts were initially screened for relevance, followed by assessment of the full text of potentially eligible publications. Duplicate records identified across databases were removed before final study selection.

The flow of records through identification, screening, eligibility assessment and inclusion is shown in Figure 1, which was adapted from the PRISMA 2020 flow diagram (28). Although this is a structured narrative rather than a systematic review, the diagram was used to make the selection process transparent. The date of the last search was 30th September 2026. Full-text records that were excluded, with reasons, are listed in Supplementary File 1.

**Figure 1:**
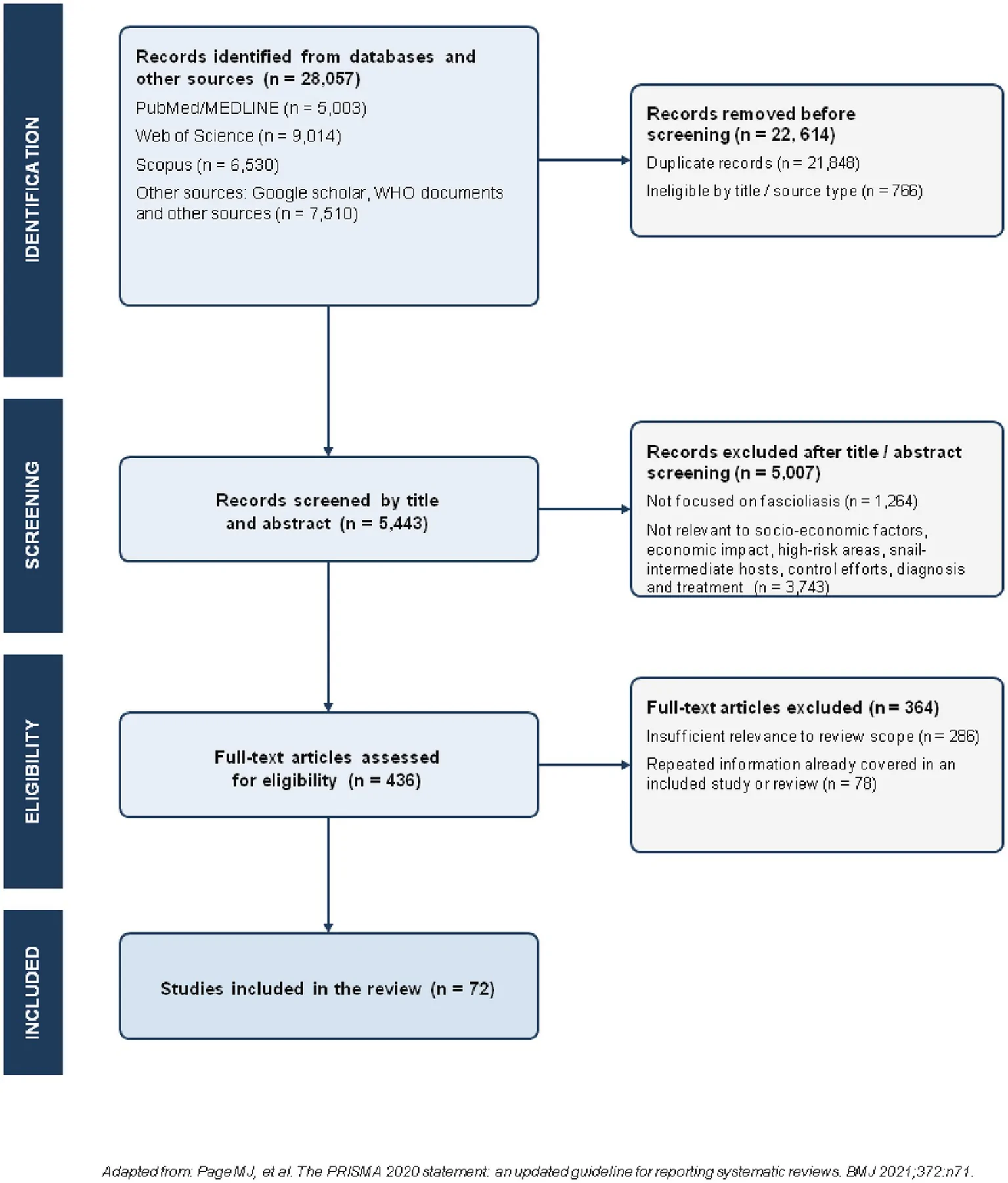
Flow diagram of literature identification, screening, eligibility assessment and inclusion for the structured narrative review. Adapted from Page et al. (28).

### 2.3 Data extraction and evidence synthesis

Information extracted from the included literature comprised publication year, study location, *Fasciola* species, fascioliasis in Kenya, study design, population or experimental system, intervention or diagnostic approach, sample size where applicable, principal outcomes, major limitations and relevance to fascioliasis in Kenya. For diagnostic studies, particular attention was given to sensitivity, specificity, reference standards, specimen type and performance at low infection intensities. For prevention and treatment studies, evidence on efficacy, safety, coverage, re-infection and implementation was considered. For emerging technologies or prevention approaches, the stage of development and extent of field or clinical validation were also evaluated.

Because the included studies encompassed heterogeneous interventions, diagnostic platforms, populations and outcome measures, no quantitative meta-analysis was undertaken. Evidence was instead synthesised narratively and critically compared across studies. Particular emphasis was placed on distinguishing established interventions from technologies undergoing field evaluation, clinical development or preclinical investigation, and on assessing their potential contribution to the transition from morbidity control towards interruption of fascioliasis transmission in Kenya. Most administrative units in this study are regarded using the old administrative style (using names such as provinces, districts and divisions) as a large percentage of the studies in this paper were conducted before the promulgation of the new Constitution in 2010 when there was the creation of ‘Counties’ as administrative units.

The first scholarly article on Fascioliasis in Kenya was published in 1960 and it was authored by Froyd. G and it was titled ‘The Incidence of Liver Flukes (*F. gigantica*) and Hydatid Cysts (*Echinococcus granulosus*) in Kenya Cattle.’

### 2.4 Quality appraisal of included studies

The methodological quality of the included primary studies was appraised by VM and GD independently, with disagreements resolved by discussion. Prevalence and abattoir-based studies, which make up most of the evidence, were appraised with the Joanna Briggs Institute critical appraisal checklist for prevalence studies (29); other designs were appraised with the JBI checklist matching their design. Each study was classified as having low, moderate or high concern about risk of bias. Reviews and WHO or regulatory documents were not scored, but their source and date were recorded. Appraisal was used to qualify the strength of the evidence in the synthesis and not to exclude studies. SANRA (27) was used only to guide the reporting quality of this review.

## 3 Results

### 3.1 Study selection

The searches of PubMed/MEDLINE (*n* = 5,003), Web of Science (*n* = 9,014) and Scopus (*n* = 6,530), together with Google scholar/WHO/other sources (*n* = 7,510), identified 28,057 records (Figure 1). Before screening, 22,614 records were removed: 21,848 duplicates and 766 that were clearly ineligible by title or source type. The remaining 5,443 records were screened by title and abstract, and 5,007 were excluded (were not focused on fascioliasis and not relevant to socio-economic factors, economic impact, high-risk areas, snail-intermediate hosts, control efforts, diagnosis and treatment of the disease). Full texts of 436 articles were assessed for eligibility, and 364 were excluded (286 with insufficient relevance to the review scope, 78 that repeated information already covered by an included study or review). In total, 72 studies were included in the review, as shown in Figure 1. The full-text articles that were excluded are listed in Supplementary File 1.

### 3.2 Characteristics of the included studies

Of the 72 included records, 70 were primary research articles and 2 were systematic or narrative reviews. Publication dates ranged from 1960 (30) to 2025 (31). 50 studies were published before 2010 and therefore use the former provincial and district boundaries. Studies were conducted in 7 of Kenya’s former eight provinces; 28 studies involved cattle, 23 studies involved sheep, 16 studies involved goats, 2 studies involved pigs, 5 studies involved wildlife, 15 studies involved snails and 2 studies involved humans.

### 3.3 Quality of the evidence

Of the 72 primary studies appraised, 70 were judged to have low concern about risk of bias, 2 moderate and 0 high. The most common limitations were convenience or abattoir-based sampling that does not represent the wider population, lack of a confirmatory diagnostic test, small sample size, and inadequate description of the sampling frame. Studies with high concern are flagged in Table 1 and are treated with caution in the discussion.

**Table 1:** Apportionment of included studies to the review themes.

Theme (Section)	No. of studies	Main study types	Studies with high concern about bias	Key references
Socio-economic factors (4.1)	[4]	[3]	[0]	(25, 34)
Economic impact (4.2)	[23]	[20]	[1]	(3, 36, 39)
Snail intermediate hosts (4.3)	[21]	[6]	[0]	(53, 56, 57)
High-risk areas (4.4)	[32]	[27]	[0]	(3, 20, 39)
Wildlife ruminant hosts (4.5)	[6]	[5]	[0]	(31, 76, 78)
Diagnosis (4.6)	[13]	[10]	[0]	(25, 82, 83)
Control efforts (4.7)	[9]	[8]	[0]	(2, 87, 88)
Treatment (4.8)	[13]	[12]	[1]	(32, 45, 98)

### 3.4 Evidence synthesis by theme

The included studies are grouped under the eight themes of the review in Table 1. The themes with the most studies were: high-risk areas (32 studies), followed by economic impact (23 studies), snail intermediate hosts (21 studies), diagnosis (13 studies), treatment (13 studies), control efforts (9 studies), wild-ruminant hosts (6 studies) and socio-economic factors (4 studies). Human fascioliasis was addressed by 2 studies. Below is a one-line summary of each theme; the evidence is discussed in Section 4.

- *Socio-economic factors*. Poverty, rural residence and water projects such as dams and irrigation schemes were associated with fascioliasis.
- *Economic impact*. Liver condemnation at slaughter was the main documented loss, followed by treatment and veterinary costs and reduced livestock productivity.
- *Snail intermediate hosts*. *Radix natalensis* and *Galba* spp. were the main snail hosts reported.
- *High-risk areas*. The former Western province had the highest reported prevalence, mostly in cattle.
- *Wildlife hosts*. Infection was reported in elephants, African buffaloes and camels only.
- *Diagnosis*. Coprological sedimentation was the main method, with little use of immunological or molecular tests.
- *Control*. Reported measures were improved husbandry, clean pasture, routine deworming and snail control.
- *Treatment*. Triclabendazole was the main drug, with plant anthelmintics as a growing alternative.

Some studies address more than one theme, so the row totals may exceed the number of included studies.

## 4 Discussion

### 4.1 Socio-economic factors

Poverty and rural communities are the two major socio-economic factors that were found to be associated with fascioliasis and other neglected tropical diseases in Kenya with the disease being shown to disproportionately affect impoverished and rural communities that have limited access to clean water and healthcare services (25, 32–34).

Rural communities tend to heavily rely on untreated water that is often contaminated with livestock or human faces. Poverty and lower household income means that households are unable to treat drinking and cooking water that they fetch from unclean water sources (17).

Water projects such as dams and irrigation schemes that are mostly set-up in rural communities significantly alter local ecosystems and create ideal breeding grounds environments for the habitation of snail intermediate hosts responsible for the transmission of Fascioliasis. More water projects mean more suitable environments for the habitation of the snail intermediate hosts hence heightened transmission of the disease in Kenya as shown in Ahero irrigation scheme and Perkerra irrigation scheme (25). Livestock farmers in Trans-Nzoia West attested to grazing their animals in swampy areas (a major risk factor in the transmission of fascioliasis) with 80.0% of the farmers not knowing the underlying cause of the disease (33).

### 4.2 Economic impact of fascioliasis in Kenya

Liver condemnation due to *Fasciola* (liver fluke) infections represents a severe drain on the agricultural economy, causing massive financial losses in Kenya. Regional and local studies by researchers like Froyd (30), Ogambo-Ongoma (9), Preston and Castelino (35), Cheruiyot (36), Nginyi et al. (37), Maingi and Gichingi (38), Kithuka et al. (3), Njeruh et al. (39), Ngaira (40), Mutua et al. (41) and Odede (42) have highlighted substantial direct financial impacts, contributing to broader figures that scale up to over £7 million annually across various regional estimates (32). Kithuka et al. (3) tracked a downward economic trend throughout the 1990s, due to routine liver condemnation in Kenyan slaughterhouses and found the estimated economic losses to be approximately US$2.6 million. (43) calculated and established economic losses of Kshs 3.505 million in condemned livers of livestock due to *F. gigantica* infections in Taveta division abattoirs, from 1989 to 2004. A study by (33) found that 88.9% of meat sellers in Trans-Nzoia West were aware that *Fasciola* infestations of livestock livers caused financial losses as a result of the infested livers being condemned. A study conducted in Kinango, Kwale by Odede (42) shows that liver condemnation in slaughterhouses within the study area caused direct economic loss of approximately US$ 20,571 during the study period.

Fascioliasis also significantly inflates agricultural operating costs through several financial drains such as direct treatment costs (farmers face high expenses for anthelmintic drugs (flukicides) to treat infected herds); veterinary fees (managing severe outbreaks requires professional veterinary care, diagnostics, and supervision) and increased labour (herd management becomes more labour-intensive due to isolation, dosing schedules, and extra animal care) as described by Kithuka et al. (3).

Chronic fascioliasis causes major economic losses in livestock through reduced productivity, lower milk and meat yields. Infected animals experience slow growth, poor weight gain, and lower meat quality. Dairy livestock produce significantly less milk, impacting farm revenues. Severe cases can lead to animal death, lower birth weights, and reduced reproductive capacity (20, 44). Fascioliasis also causes abortions, miscarriages, infertility, stunted growth, pre-disposition to other infections, reduced milk production, reduced meat production and high mortalities in calves and ruminants (10, 43, 45–49).

### 4.3 Snail intermediate hosts

Snail intermediate hosts are vital for the transmission of fascioliasis, with *Fasciola* parasites utilising snail intermediate hosts to complete their life cycle. Asexual stages of *Fasciola* parasites in Kenya often utilise *Radix natalensis* and *Galba* spp. snails (9, 23, 34, 50–54). Most intermediate snails are often found attached to vegetation, in slow flowing, shallow waters rich in organic waste (34, 53, 55). *Radix natalensis* snails in Kenya also have a preference for irrigation schemes, stagnant freshwater habitats and swamps (53, 56).

Water velocity has been cited as the most important factor in lotic environments that influences the distribution of *Radix* spp. in the Mara river basin (53). High snail densities have been found to typically concentrate a short distance from the source of rivers or along slow-moving spring offshoots right before they join main river channels (57). Temperature also plays an important role in the distribution of *Radix* and *Galba* snails in Kenya but it appears to play a more important role in lentic environments than in lotic environments (53). Temperature has also been shown to affect the development of cercariae from the rediae, with temperatures less than 16 °C discouraging the production of cercariae from the rediae which normally from the sporocyst while in the snail host (58). Rainfall has also been shown to be vital in the occurrence and abundance of *Radix natalensis*, with the greatest increase in numbers of the snails occurring during the rainy season (20, 52), while a study by Teesdale (59) found little seasonal change in the populations. Organic matter, fuelled by human and animal excreta has been shown to promote the growth and abundance of algae, with algae being regarded as one of the best food for snails (53). Vegetation is also important as a medium of laying eggs for the snails, an attachment substrata (helps them rest, avoid being swept away by water flow) and also a source of food (53, 57). pH has been shown to have a role on the occurrence and abundance of *Radix natalensis* with Preston and Castelino (35) asserting that laboratory tests indicate that *Radix natalensis* is highly susceptible to alterations in pH, with a change from 7·0 to 6·0 resulting in 90% mortality. Reduced photosynthetic activity of water flora, degradation of organic matter, disturbance of salts on the dam bed and leaching of various salts, especially sulphides, are known to effect pH changes (60, 61);

*Radix natalensis* have been found in the Mara River Basin (53), in informal settlements of Kisumu (34), in the former Western province (55), in Taita-Taveta County (in streams connecting to Lumi River, in irrigation furrows with water sources same to Lumi River and in Lake Jipe) as described by Hisatake et al. (62). A study by Cheruiyot and Wamae (57) also found the snails in Molo, Eldoret, Londiani and Karatina.

### 4.4 High risk areas

High risk areas (areas with the highest prevalence of fascioliasis) are those that experience high rainfall and harbour permanent water bodies (lakes, rivers, streams, ponds) or irrigation schemes. The World Health Organisation asserts that warmer temperatures and increased rainfall are conducive for the spread of fascioliasis (17). High infection levels are frequently reported in Western Kenya, the Rift Valley (including Narok and Baringo), the Lake Victoria basin (such as Ahero), irrigation zones like Perkera and parts of the Coastal strip that experience enhanced rainfall throughout the year and favour the survival of the intermediate host, *Radix natalensis* (26, 53). It has also been shown that in Kenya, fascioliasis is more prevalent in swampy areas (25). The breed and age of livestock, tentatively has a role to play in their susceptibility to *Fasciola* infections with indigenous African breeds shown to be more resistant to infections compared to exotic breeds, native to Europe and older cattle appear to be more susceptible to infections (63–65). We speculate that prevalence increased with age because of the longer period in which older animals are exposed to infected pastures.

A 10 years (1990–1999) retrospective study by Kithuka et al. (3) of abattoir data from Dagoretti slaughterhouses, one of the largest slaughterhouse complex in Kenya with cattle being sourced from all parts of the country established the highest prevalence of bovine fascioliasis in cattle from the former Western province (16%) followed in descending order by Eastern Province (11%), Nyanza Province (9%), Rift Valley Province (8%), Central Province (6%), Nairobi Province (4%) and Coast Province (3.5%); despite majority of the cows originating from Rift Valley Province. In the 10 years (1990–1999) retrospective study by Njeruh et al. (39) using post mortem records of cattle, sheep and goats collected at the Veterinary Department Headquarters Kabete (with records from abattoirs in 38 districts in 8 provinces of Kenya), the prevalence rate in Western province for cattle, sheep and goats was 16%, 10 and 9%, respectively, and the lowest prevalence of *Fasciola* infection was in Coast Province (3.5% for cattle, 0.74% for sheep and 0.5% for goats).

Western province has been found to be the province with the highest prevalence of fascioliasis, in most studies, with an average bovine prevalence of above 15% as shown in Figure 2.

**Figure 2:**
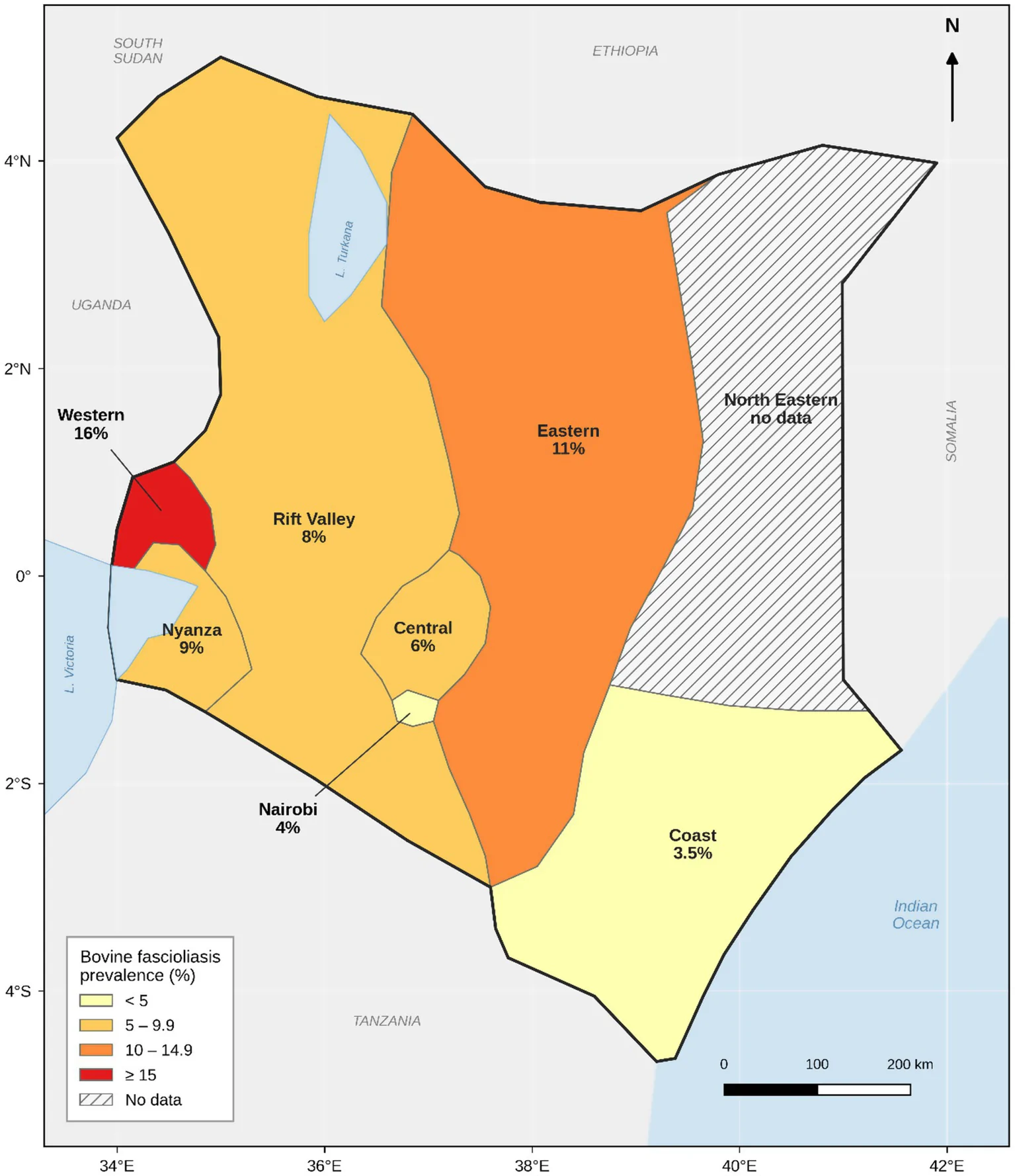
Kenya’s bovine fascioliasis by provinces; map was created using R version 4.6.1.

The high prevalence of bovine fascioliasis in the former Western province of Kenya and this has been attributed to high rainfall (approximately 1,200–2,200 mm) and the high presence of *Radix natalensis* in the region, necessitated by favourable climatic conditions that support the abundance of the snails (3, 39). A study in Busia district by Kagira et al. (66) established a fascioliasis prevalence of 3% in pigs whereas an earlier study by Karanja (46) in the same Busia district had established a prevalence of 22% in pigs. The difference in prevalence in Busia district is likely due to the fact that the Karanja (46) study investigated fascioliais prevalence in free-range scavenging pigs that had greater access to wet, marshy areas containing the intermediate host snails (*Lymnaea* species) that transmit liver flukes whereas the Kagira et al. (66) study investigated fascioliais prevalence in pig farms with improved smallholder management, restricted free-range grazin and better targeted veterinary interventions or deworming practices.

Nyanza province has been reported to exhibit high prevalances of fascioliasis in domestic ruminants with acute fascioliasis caused by *F. gigantica* infections in small ruminants (3, 24, 67). A study by Wachira and Shavulimo (67) found that helminthosis (including fascioliasis) was the second most important cause of mortality, after pneumonia, in cross-bred exotic goats in Kisumu district. The retrospective study by Kithuka et al. (3) on the other hand found prevalence of bovine fascioliasis in Nyanza Province to be 9%. A study by Kanyari et al. (24) that investigated the prevalence and intensity of endoparasites in 66 sheep and 60 goats kept by urban and peri-urban farmers in Kisumu Municipality found 37% *Fasciola* infection rate in sheep and 36% *Fasciola* infection rate in goats. The high prevalence of fascioliasis in Nyanza province could be attributed to the availability of many swampy and muddy areas in the province, that are ideal for the habitation of snail intermediate hosts of *F. gigantica* (34, 55).

Eastern province has also been reported to prevalent with fascioliasis, as reported by Ogambo-Ongoma (9), Kithuka et al. (3) and Waruru et al. (68). A 1969 study by Ogambo-Ongoma (9) with surveys conducted at the Athi River abattoir discovered 30.7% (190 out of 618 cattle livers) infected with *F. gigantica* and 2 of the livers infected with *F. hepatica*. The retrospective study by Kithuka et al. (3) of abattoir data from Dagoretti slaughterhouses established the bovine fascioliasis of livestock sourced from Eastern Province to be 11%. On the other hand, a study by Waruru et al. (68) of small ruminants conducted on two farms in Kathiani Division of Machakos District, Kenya for 18 months found an overall prevalence of liver flukes (*Fasciola* infections) to be 31.5%. The variance between the studies in Eastern province (11 to 31.5% prevalence) highlights how prevalence rates can shift depending on the specific district, environment, climatic factors, and whether the data comes from a local farm or a centralized slaughterhouse (3, 9, 68).

Central province has been found prevalent with fascioliasis as demonstrated in (38, 39, 69). A study conducted on 16 dairy cattle farms in Kiambu district from 1991 to 1992 reported a prevalence of 34% *F. gigantica* infection among the cattle, with prevalence higher in older cattle than younger cattle (69). The higher prevalence observed in older cattle in Kiambu District (34%) aligns with the chronic, cumulative nature of *F. gigantica* exposure, where older grazing animals accumulate larger fluke burdens over time. A study conducted in Nyandarua district by Maingi and Gichingi (38), between 1994 and 1995 from 30 slaughter slabs established a prevalence of 15.5% among cattle and sheep in the district. *Fasciola* spp. infections in sheep domiciled at small scale farms in Central Kenya varied from 0 to 5.1%, in a study conducted between 1997 and 1998 (70). The variation in prevalence between districts—from 34% on dairy farms in Kiambu down to 15.5% at slaughter slabs in Nyandarua—highlights how localized microclimates, irrigation, and proximity to marshy snail habitats influence parasite transmission dynamics. The lower prevalence range in small-scale sheep farms (0 to 5.1%) compared to local cattle suggests differing grazing management practices, as smallholder sheep are less likely to access the marshy water sources or swampy pasture communal zones where intermediate snail hosts that shed *Fasciola* mutlicercariae thrive.

Rift valley province has been reported to be prevalent with fascioliasis, courtesy of several studies (33, 37, 40, 54, 71–73). An examination of human stool from natives of the Lake Baringo area established a 3% *F. hepatica* incidence of infection (71). A study conducted in the Trans-Mara Division of Narok District found Liverfluke *F. gigantica* infestation throughout the Division and about 50% of faecal samples from cattle, sheep and goats were positive for *Fasciola* eggs (72). Central Rift Valley has also been found prevalent with Fasciolosis, primarily driven by *F. gigantica* (54). A study at 2 abbatoirs in Kajiado district established a *F. gigantica* prevalence of 0.3% in the sheep and 0.3% in the goats slaughtered, over a 6 months period (37). A 5 years (2011–2015) retrospective study in Trans-Nzoia County established a 5.85% fascioliasis prevalence among cattle, sheep and goats slaughtered in the county with cattle, sheep, and goat recording individual prevalence of 6.52%, 6.08 and 4.10%, respectively, (33). A 2016 study by Ngaira (40) carried out in Nandi Central (Nandi County) with secondary data was collected from Kapsabet District Veterinary Office, found out that 65.2% of the condemned livers in abattoirs were from cattle, 30.9% from sheep and 3.9% from goats. A study conducted between 2016 and 2017 in Kerio Valley (Elgeyo Marakwet County) established an overall Fascioliasis prevalence of (17.3%) for all the sampled livestock, with 14.5% of cattle infected, and 9.76% of sheep infected (73). Lastly, a study by Wafula (74) on goats from Trans Nzoia County established a Fascioliasis prevalence of 14.5%, with rainy seasons having a higher prevalence of gastro-intestinal helminth infections. The difference in fascioliasis prevalence in Rift Valley province is attributable to differences in host susceptibility to *Fasciola* infections with cattle regularly displaying the highest rates of infection and accounting for the vast majority of condemned livers in regional abattoirs, though goats and sheep remain vulnerable, especially during high-rainfall periods. Seasonality is the other factor that contributes to varying prevalence of *Fasciola* infections in Rift Valley province with rainy seasons presenting a significantly higher risk due to the proliferation of intermediate snail hosts (33, 37, 40, 54, 71–73).

Irrigation schemes and permanent watersheds have also been shown as high risk areas for the transmission of fascioliasis in Kenya (25). A recent bovine fascioliasis study conducted at Ahero irrigation scheme (Kisumu county), Mara river basin (Narok county) and Perkera irrigation scheme (Baringo county) established a prevalence of 35.1%, 27.0 and 38.0%, respectively, with the irrigation schemes in Kisumu and Baringo counties believed to play an important role in providing marshy areas and vegetation for the breeding of the snail intermediate hosts and attachment of *Fasciola* metacercariae (25).

Coast Province has been reported to be prevalent with fascioliasis, albeit at a significantly low rate compared to other provinces; this has been attributed to the fact that Coast Province receives relatively low rainfall compared to the other provinces and this is not conducive for the abundant habitation of the snail intermediate hosts that support transmission (3, 26, 36). A study by Mungube et al. (43) in the semi-arid coastal Kenya study area (Taveta division), between 1989 and 2004 demonstrated *F. gigantica* infection rates of 26%, 6.6 and 5.2% in cattle, goats and sheep, respectively. A cross-sectional study conducted by Mungube et al. (26) in selected settlements of Taita-Taveta County established overall fasciolosis prevalence in cattle, goats and sheep to be 25.9%, 23.4 and 33.3%, respectively. A study by Cheruiyot et al. (36) found the prevalence of fascioliasis in bovines slaughtered in the abattoirs of Coast Province of Kenya to range from 0 to 8%. A retrospective study by Odede (42) on slaughterhouse records of cattle from Kinango Sub-County, Kwale County established a 2.8% prevalence rate based on 1,600 condemned livers out of 57,577 cattle slaughtered.

### 4.5 Wildlife ruminant hosts

Wildlife hosts populations play the role of reservoir hosts and can maintain and cycle liver fluke infections independently of domestic livestock, presenting challenges for disease control in mixed wildlife-livestock ecosystems (75). In Kenya *Fasciola* infections have been found in elephants, African buffaloes and camels (31, 76–78).

Elephants domiciled at Tsavo East National Park (TENP) and the Laikipia Samburu Ecosystem (LSE) were found infected with *Fasciola* parasites, with trematodes infection prevalence recorded as 39.4 and 25.7%, respectively. The presence of *Fasciola* parasites in the two ecological zones (TENP and LSE) could be attributed to the presence of marshes, swamps, streams, and even possible watering holes that provide suitable environments for the intermediate snail hosts (31). A study by King’ori et al. (76) on elephant populations in Maasai Mara National Reserve, Tsavo East National Park, Amboseli National Park and Laikipia-Samburu Ecosystem found the elephants infected with trematodes (*Protofasciola* and *Brumptia*) at a prevalence of 39.1%; this study does not report the availability of *Fasciola* parasites. *Fasciola hepatica* has been demonstrated as the parasite that mostly infects elephants in Kenya and not *F. gigantica*; with *Fasciola jacksoni*, a notorious *Fasciola* species, unique to elephants and a critical threat to Asian elephants in countries like Sri-Lanka and India, not yet reported in Kenya (31, 79, 80). The phenomenon of Kenyan elephants (*Loxodonta africana*) primarily showing infections of *F. hepatica* despite *F. gigantica* being the most common liver fluke in the country comes down to a mix of ecological zoning, intermediate host constraints, and dietary habits. *Fasciola hepatica* is highly adapted to cooler, temperate zones and the wet highlands of Kenya. This geographical overlap is crucial because key elephant populations frequently range through or retreat into these cooler, highly forested montane ecosystems (like Mt. Kenya, the Aberdares and Mt. Elgon). In addition, *F. hepatica* relies on amphibious *Lymnaea* (such as *Galba/Fossaria*) snails that thrive in the mud and cool water bodies of highland zones. Elephants foraging in highland water sources, marshes, and damp forest floors are far more likely to encounter vegetation contaminated with the infective stage (metacercariae) of *F. hepatica* shed by these highland snails (21, 79).

African buffaloes in Kenya have been shown to be highly infected with *F. gigantica* courtesy of extensive wildlife surveys with liver flukes found in the bile ducts or gallbladders of approximately 50% of African buffaloes in certain ecosystems. Buffaloes pick up the infection in marshy or wetland habitats where mud snails act as intermediate hosts, releasing parasite larvae that encyst on aquatic vegetation (77).

Camels found in Kenya have also been shown to be infected by *Fasciola* infections (78). A study by Mukhwana (78) found camels in Samburu District infected with *Fasciola* parasites after examination of 255 camels’ faecal samples taken over a period of 5 months with the faecal samples being subjected to the McMaster egg counting technique and coproculture.

### 4.6 Diagnosis of fascioliasis in Kenya

Diagnosis of fascioliasis in Kenya primarily relies on coprological (stool/faecal) sedimentation techniques to detect *Fasciola* ova in livestock, while veterinary abattoir inspections and clinical evaluations support regional surveillance (25, 69, 74, 81). The sedimentation technique has for a long time been used as the diagnostic technique for diagnosing fascioliasis in Kenya, as indicated by reports from Kenya Agricultural Research Institute, the International Livestock Research Institute and other organisations, including universities (25, 26, 42, 74, 82, 83).

The diagnosis of food-borne trematodiasis traditionally relies on the parasitological examination of faecal samples in search of the parasites’ eggs, with coprological analysis being the common used method (25). Coprological analysis is crucial because it provides important information that helps identify the number of infected individuals releasing eggs (84). However, traditional coprological analysis has low analytical sensitivity, misses low burden infections and fails to detect pre-patent and sub-patent infections before eggs are actively shed (17, 84). Coproantigen tests can be used to overcome the low sensitivity of coprological analysis by detecting parasite-specific proteins directly in faecal samples (17, 85). However, currently, there is a dearth of published literature regarding the use of coproantigen tests in Kenya and this suggests that the coproantigen tests are yet to be widely embraced in Kenya and these could be due to systemic and infrastructural barriers.

### 4.7 Control efforts

Interrupting the transmission of fascioliasis (liver fluke infection) in Kenya has been shown to require a multi-pronged approach targeting both the definitive livestock hosts and the intermediate snail hosts. Because the disease is heavily dependent on wet environments like wetlands, riverbanks, and flooded pastures, breaking the lifecycle relies on combining these key husbandry and medical interventions (2, 26, 86–88).

Improved animal husbandry practices, transitioning toward intensive production systems or zero-grazing prevents livestock from roaming into contaminated wetlands interrupts transmission. Feeding livestock clean, sun-dried pasture or hay harvested away from water edges significantly eliminates viable metacercariae (infectious larvae). Restricting livestock from drinking directly from stagnant or slow-moving natural water bodies removes a major infection source, complemented with providing safe water at home via clean troughs breaking direct contact with *Fasciola* parasites (2, 26, 47, 72).

Intermediate snail hosts control is one of the approaches used to control fascioliasis in Kenya. Use of molluscicides to kill the snails, biological control of the snails by using competitor snails and environmental modification of snail habitats are some of the approaches that can be used to thwart the transmission of fascioliasis with the use of with synthetic molluscicides such as N-trityl morpholine and copper sulphate being the synthetic molluscicides of choice (35, 87, 88). Elimination of snail intermediate hosts from the environment implies that *Fasciola* parasites will not complete their life cycle and therefore new transmission will be thwarted. A study in a Nandi estate by Preston and Castelino (35) used N-tritylmorpholine (a synthetic molluscicide) to eliminate snail intermediate hosts of several parasites (including *Bulinus tropicus, Biomphalaria pfeifferi* and *Radix nastalensis*) in nearby dams, with consequential positive effects on the health of livestock in the study region (including weight gain, better carcass conformation with completely clean livers—not infected with liver flukes); suggesting killing of the snail intermediate hosts interrupted the transmission of fascioliasis and in the process, the number of *Fasciola* infections significantly declined. The primary active ingredients of synthetic molluscicides are synthetic compounds like Metaldehyde, Niclosamide, and Copper Sulfate. Synthetic molluscicides function by destroying mucus-producing cells causing severe dehydration, or disrupting cellular respiration. However, synthetic molluscicides have several drawbacks, such as being unsafe to the environment (having a higher environmental persistence, resistant to rapid degradation) and killing non target organisms (frequently toxic to fish, amphibians, beneficial soil invertebrates, and mammals). Synthetic molluscicides are also associated with resistance development in target organisms as the organisms quickly adapt to single, highly concentrated synthetic molecules.

Plant molluscicides have been embraced in recent years in Kenya, for snail control purposes due to having been shown to be effective in reducing snail populations and to be less harmful to the environment, as well as being generally safe non-target hosts such as mammals. The primary active ingredients of plant molluscicides are natural secondary metabolites like saponins, tannins, alkaloids, and flavonoids. Saponins have although been reported to be highly toxic to fish. The mode of action of plant molluscicides is by disrupting cell membranes (saponins), causing neurotoxicity, or acting as a feeding deterrent. Resistance development to plant molluscicides by target organisms has been reported to be slow when compared to resistance development to synthetic moluscicides by the target organisms. Plant molluscicides have also been shown to quickly break-down in nature (possess high biodegradability) and given that plant molluscicides utilise local resources (common trees, weeds and crops), they eliminate the economic need to import synthetic molluscicides (87). *Glinus lotoides* extracts have been used in Kenya as an effective plant molluscicide against intermediate hosts and parasites of *Fasciola* and *Schistosoma* because of their strong molluscicidal (snail-killing) and cercariacidal (larvae-killing) properties (86). While plant-based molluscicides offer significant environmental advantages, they generally fall short of the rapid, high-kill potency seen in synthetic molluscicides.

The use of competitor snails such as *Marisa cornuarietis* (the giant ram’s horn snail), *Melanoides tuberculata* and *Planorbis planorbis* to displace vector snails has successfully been demonstrated in other parts of the world (89, 90). While these historical biological control programs primarily targeted *Biomphalaria* or *Bulinus* snails to halt the transmission of human schistosomiasis (bilharzia), the ecological principle remains the same. In these regions, the competitor snails effectively suppress vector populations through intense competition for food and space, as well as by accidentally consuming the egg masses of the host snails. Tanzania directly proved that introduced populations of *Marisa cornuarietis* completely eliminated *Radix natalensis* (the primary intermediate host for the liver fluke *F. gigantica*, which causes fascioliasis) from local freshwater habitats (90). Despite its proven efficiency in suppressing *Radix natalensis* populations elsewhere, this biological management strategy has not been implemented or documented for controlling livestock fascioliasis transmission within Kenya.

However, from an environmental perspective, killing mud snails (*Radix* spp. and *Galba* spp.) would cause a significant, negative disruption to their native environments and ecosystems. If *Radix* spp. and *Galba* spp. were completely removed, the ecosystem would experience cascading impacts across several categories such as breakdown of the aquatic food web (these snails offer calcium-rich food source for many native species and also offer significant energy transfer to higher trophic levels). Elimination of mud snails from the environment would also lead to an explosion of algae and organic waste as mud snails are essential grazers that primarily consume algae, biofilm, and detritus and this will consequentially lead to water quality deterioration from the algal blooms (91, 92).

### 4.8 Treatment of fascioliasis in Kenya

Use of anthelmintics, specifically Triclabendazole, the drug recommended by the World Health Organisation in the treatment of fascioliasis at a dosage of 10 mg/kg (17) is one of the most common approaches used in the control of fascioliasis in Kenya (26, 47). Triclabendazole is typically administered orally with adults taking a single dose per day and children aged 6 and older, two doses per day (17, 93). Maingi et al. (93) suggest that for optimal efficacy of Triclabendazole in livestock, treatments should be given during the dry seasons as treatment during the rainy seasons is likely to be ineffective. It has been ascertained that Triclabendazole treatment plays an important role in improving fertility, higher birth weights and improved meat and milk production of sheep in Kenya (93). Strategic use of separate anthelmintic treatments for *Fasciola* and gastrointestinal nematodes (Triclabendazole, at 10 mg/kg body weight targeted *Fasciola* parasites, while levamisole, at 10 mg/kg body weight targeted nematodes); on a farm in the Kenya highlands yielded the highest birth weights and weight gains in sheep suggesting that combined treatments administered at a single time, at the beginning of the wet season are better than single treatments when dealing with co-infections (93).

The high cost and limited accessibility of Triclabendazole are critical bottlenecks that hinder large-scale drug administration programs to control and eliminate fascioliasis (liver fluke disease) in Kenya. Because Triclabendazole is the only World Health Organization (WHO)-recommended drug effective against both the immature and mature stages of the parasite, dependency on it creates severe economic and programmatic vulnerabilities. Beyond the drug’s price tag, fully curtailing the disease is complicated by the presence of mud snails (the parasite’s intermediate host) in marshy areas, agricultural projects, and communal watering holes create constant re-infection pressure (94). Resistance to Triclabendazole by *Fasciola* parasites continues to a growing global concern in the fight against the disease (95).

The potential use of plant anthelmintics for the control of livestock helminthoses (including fascioliasis) in Kenya continues to be explored because of costs associated with Triclabendazole and potential development of anthelmintic resistance (96–98). Some of the plant anthelmintics that have been experimentally tested for their efficacy against fascioliasis include *Annona squamosa, Ananas comosus* (97); *Albizia anthelmintica* (99); *Albizia anthelmintica*, *Myrsine Africana* (98); *Allium sativum*, *Allium cepa* (100). Studies on potential use of plant anthelmintics in Kenya are ongoing with experiments being carried out to determine efficacy, toxicity and optimum dosage rates (96, 101).

### 4.9 Limitations of the study

The evidence base is dominated by abattoir and field surveys in cattle, many conducted before 2010 with varied diagnostic methods and sampling, and there are almost no data on human infection. Prevalence figures should therefore be read as indicative, not as directly comparable estimates.

## 5 Conclusion

In conclusion, fascioliasis is a disease of public health, veterinary health, wildlife health and socio-economic importance in Kenya. *Fasciola gigantica* is the most important *Fasciola* parasite, responsible for the transmission of fascioliasis in Kenya, with *F. hepatica* infections recorded in a few places. Most *Fasciola* infections in Kenya have been reported in cattle, goats and sheep; with few reports of *Fasciola* infections in elephants, African buffaloes and camels. Currently, there is no official data on human fascioliasis in Kenya.

Economic losses that stem from fascioliasis occur when *Fasciola* infected livers are condemned in slaughterhouses of Kenya with estimates of more than £7 million financial losses annually. Fascioliasis also increases the operational cost of running an agri-business enterprise as it instigates an increase in the cost of animal treatment as farmers attempt to treat the disease Fascioliasis causes abortions, miscarriages, infertility, stunted growth, pre-disposition to other infections, reduced milk production, reduced meat production, slow growth, weight loss, low quality of milk, low quality of meat and high mortalities. Socio-economic factors associated with fascioliasis include poverty (lower-household incomes), water projects (development of dams and irrigation schemes) and rural communities.

Control efforts to reduce the prevalence of fascioliasis in Kenya, often target the snail intermediate hosts (to stop *Fasciola* parasite from completing its life-cycle), by the use of synthetic molluscicides such as N-tritylmorpholine and Copper Sulphate to kill the snails. However, because of drawbacks associated with synthetic molluscicides such as being unsafe to the environment and killing of non target species, plant molluscicides are gradually being embraced as an alternative. The advantages of plant molluscicdes to synthetic molluscicides include being less harmful to the environment, not killing non-target species, quick break-down in nature, eliminating the economic need to import synthetic molluscicides and utilising of local resources. The standard method used to diagnose fascioliasis in Kenya is coprological examination (sedimentation technique) where faecal specimens from humans and ruminants are analysed.

High risk fascioliasis transmission areas in Kenya have been ascertained and these are: Western Province, Nyanza province, Eastern province, Central Province and Rift Valley molluscicides Province with Western province consistently leading in prevalence and Coast province being the province with the least prevalence in nationwide studies, possibly because climatic and environmental conditions in the Coast province do not favour the abundant distribution of *Radix* spp. and *Galba* spp., two important genera necessary for the transmission of the disease. In addition, marshy areas, irrigation schemes and stagnant freshwater habitats and swamps have been shown to be ideal for the habitation of *Fasciola* spp. snail intermediate hosts.

Treatment of *Fasciola* infections in Kenya, as per the World Health Organisation recommendation involves oral administration of Triclabendazole—an anthelmintic at a dosage of 10 mg/kg. Experimental dosage formulations suggest that Triclabendazole treatments in livestock should be given during dry seasons as treatment during rainy seasons is likely to be ineffective. It has been proven that Triclabendazole treatment plays an important role in improving fertility, higher birth weights and improved meat and milk production of livestock in Kenya. Because of the high cost, limited accessibility and potential resistance to Triclabendazole, Kenyan researchers continue to explore the potential use of plant anthelmintics in the control of fascioliasis with plants such as *Annona squamosa, Ananas comosus*, *Albizia anthelmintica, Myrsine Africana*, *Allium sativum* and *Allium cepa* promising as ideal candidates. These studies on potential plant anthelmintics in Kenya often involve experiments that determine efficacy, toxicity and optimum dosage rates of the ethnomedicinal plants.

## References

[1] Dube A Kalinda C Manyangadze T Chimbari MJ. Effect of water temperature on life history traits of *Radix natalensis*, the intermediate host of *Fasciola gigantica*. BMC Vet Res. (2026) 22:418. doi: 10.1186/s12917-026-05550-4, 42157212 PMC13361778

[2] Kariuki DP. Mortality in young stock and diseases of specific importance in the small ruminants in Kenya. Bull Off Int Epizoot. (1980) 92:1247–51.

[3] Kithuka JM Maingi N Njeruh FM Ombui JN. The prevalence and economic importance of bovine fascioliasis in Kenya-an analysis of abattoir data. Onderstepoort J Vet Res. (2002) 69:255–62. Available online at: https://repository.up.ac.za/server/api/core/bitstreams/5708d3f1-54da-4030-8209-c69055f212b5/content 12625377

[4] Tidman R Kanankege KST Bangert M Abela-Ridder B. Global prevalence of 4 neglected foodborne trematodes targeted for control by WHO: a scoping review to highlight the gaps. PLoS Negl Trop Dis. (2023) 17:e0011073. doi: 10.1371/journal.pntd.0011073, 36862635 PMC9980766

[5] Keiser J Utzinger J. Food-borne trematodiases: current chemotherapy and advances with artemisinins and synthetic trioxolanes. Trends Parasitol. (2007) 23:555–62. doi: 10.1016/j.pt.2007.07.012, 17950667

[6] Avcioglu H Guven E Balkaya I Kaynar O Hayirli A. Evaluation of coprological and serological techniques for diagnosis of bovine fasciolosis. Isr J Vet Med. (2014) 69:203–10.

[7] Abebe R Abunna F Berhane M Mekuria S Megersa B Regassa A. Fasciolosis: prevalence, financial losses due to liver condemnation and evaluation of a simple sedimentation diagnostic technique in cattle slaughtered at Hawassa municipal abattoir, southern Ethiopia. Ethiop Vet J. (2010) 14:39–51. Available online at: http://ajol.info/index.php/evj/article/view/63868

[8] Dermauw V Muchai J Al Kappany Y Fajardo Castaneda AL Dorny P. Human fascioliasis in Africa: a systematic review. PLoS One. (2021) 16:e0261166. doi: 10.1371/journal.pone.0261166, 34882738 PMC8659297

[9] Ogambo-Ongoma AH. The incidence of *Fasciola hepatica* Linnaeus, 1758, in Kenya cattle. Bull Epizoot Dis Afr Bull Des Epizoot En Afr. (1969) 17:429–31. 5394143

[10] Hammond JA Sewell MMH. The pathogenic effect of experimental infections with *Fasciola Gigantica* in cattle. Br Vet J. (1974) 130:453–65. doi: 10.1016/S0007-1935(17)35788-3, 4434159

[11] Mas-Coma S Valero MA Bargues MD. Chapter 2. *Fasciola*, lymnaeids and human fascioliasis, with a global overview on disease transmission, epidemiology, evolutionary genetics, molecular epidemiology and control. Adv Parasitol. (2009) 69:41–146. doi: 10.1016/S0065-308X(09)69002-3, 19622408

[12] Alemneh T Ayelign M. Study on prevalence and economic importance of bovine fasciolosis in three districts of north-East Amhara region, Ethiopia. J Infect Non Infect Dis. (2017) 3:24. doi: 10.3390/tropicalmed8040208

[13] Maciel MG Lima WDS de Almeida FLM Coelho LIARC Araújo GAN Lima MG . Cross-sectional serological survey of human fascioliasis in Canutama municipality in Western Amazon, Brazil. J Parasitol Res. (2018) 2018:6823638. doi: 10.1155/2018/6823638, 29593895 PMC5822807

[14] Rehman T Khan MN Abbas RZ Babar W Sikandar A Zaman MA. Serological and coprological analyses for the diagnosis of *Fasciola gigantica* infections in bovine hosts from Sargodha, Pakistan. J Helminthol. (2016) 90:494–502. doi: 10.1017/S0022149X15000711, 26300295

[15] Hadebe MI Manyangadze T Kalinda C Mindu T Chimbari MJ. Infection rates of *Fasciola* intermediate host snail species and their distribution in Africa: a systematic review and meta-analysis. Trop Med Infect Dis. (2023) 8:10. doi: 10.3390/tropicalmed8100467, 37888595 PMC10610779

[16] Ngcamphalala PI Nyagura I Malatji MP Mukaratirwa S. Susceptibility of lymnaeid snails to *Fasciola hepatica* and *Fasciola gigantica* (Digenea: Fasciolidae): a systematic review and meta-analysis. PeerJ. (2025) 13:e18976. doi: 10.7717/peerj.18976, 40098811 PMC11913016

[17] World Health Organization (2025) Neglected tropical diseases: Fascioliasis World Health Organization. Retrieved September 9, 2026, from Neglected tropical diseases: Fascioliasis

[18] Liu M Clarke LJ Baker SC Jordan GJ Burridge CP. A practical guide to DNA metabarcoding for entomological ecologists. Ecol Entomol. (2020) 45:373–85. doi: 10.1111/een.12831

[19] Moshfe A Aria A Erfani N Jamshidi A Sarkari B Abdolahi Khabisi S . Clinical features, diagnosis and management of patients with suspicion of fascioliasis in Kohgiluyeh and Boyer-Ahmad Province, southwestern Iran. Iran J Parasitol. (2020) 15:84–90. doi: 10.18502/ijpa.v15i1.2530, 32489379 PMC7244844

[20] Bitakaramire PK. Bovine fascioliasis in Kenya. Bull Epizoot Dis Afr. (1968a) 16:107–13. 5687626

[21] Malone JB Gommes R Hansen J Yilma JM Slingenberg J Snijders F . A geographic information system on the potential distribution and abundance of *Fasciola hepatica* and *F. gigantica* in East Africa based on food and agriculture organization databases. Vet Parasitol. (1998) 78:87–101. doi: 10.1016/S0304-4017(98)00137-X, 9735915

[22] Legesse S Tsegaye S Lamesgen S Wolelaw Y Garikipati D Wondimagegn W. Coprological prevalence and associated risk factors of bovine fasciolosis in and around Zenzelma, Bahir Dar, Ethiopia. Eur J Exp Biol. (2017) 7:34. doi: 10.21767/2248-9215.100034

[23] Nukeri S Malatji MP Sengupta ME Vennervald BJ Stensgaard AS Chaisi M . Potential hybridization of *Fasciola hepatica* and *F. gigantica* in Africa—a scoping review. Pathogens (Basel). (2022) 11:1303. doi: 10.3390/pathogens11111303, 36365054 PMC9695073

[24] Kanyari PWN Kagira JM Mhoma JR. Prevalence and intensity of endo-parasites in small ruminants kept by farmers in Kisumu municipality, Kenya. Livest Res Rural Dev. (2009) 21:111–6. Available online at: https://lrrd.cipav.org.co/lrrd21/11/kany21202.htm

[25] Kipyegen CK Muleke CI Otachi EO. Human and animal fascioliasis: coprological survey in Narok, Baringo and Kisumu counties, Kenya. Onderstepoort J Vet Res. (2022) 89:e1–6. doi: 10.4102/ojvr.v89i1.1954, 35144443 PMC8831969

[26] Mungube EO Sila DM Kariuki CW Bauni SM Tenhagen BA Wamae L. (2012). A cross-sectional survey on fascioliasis in selected settlements of Taveta division, coast province, Kenya. Livest Res Rural Dev 24:58. Available online at: http://www.lrrd.org/lrrd24/4/mung24058.html (Accessed December 22, 2025)

[27] Baethge C Goldbeck-Wood S Mertens S. SANRA-a scale for the quality assessment of narrative review articles. Res Integr Peer Rev. (2019) 4:5. doi: 10.1186/s41073-019-0064-8, 30962953 PMC6434870

[28] Page MJ McKenzie JE Bossuyt PM Boutron I Hoffmann TC Mulrow CD . The PRISMA 2020 statement: an updated guideline for reporting systematic reviews. J Clin Epidemiol. (2021) 134:178–89. doi: 10.1016/j.jclinepi.2021.03.001, 33789819

[29] Munn Z Moola S Lisy K Riitano D Tufanaru C. Methodological guidance for systematic reviews of observational epidemiological studies reporting prevalence and cumulative incidence data. Int J Evid Based Healthc. (2015) 13:147–53. doi: 10.1097/XEB.0000000000000054, 26317388

[30] Froyd G. The incidence of liver flukes (*Fasciola gigantica*) and hydatid cysts (*Echinococcus granulosus*) in Kenya cattle. J Parasitol. (1960) 46:659–62. doi: 10.2307/3274957, 13702295

[31] Mogaka SK Mwangi EM Fasina FO Mutahi WW. Prevalence of helminthiasis among African elephants in Tsavo and Laikipia-Samburu ecosystems, Kenya. Vet Sci. (2025) 12:1161. doi: 10.3390/vetsci12121161, 41472141 PMC12737649

[32] Acharry G. O. (2015) A Day in the Life of the PAZ Project: Fascioliasis in Western Kenya. PAZ Project. Available online at: http://www.zoonotic-diseases.org/a-day-in-the-life-of-the-paz-project-fascioliasis-in-western-kenya/ (Accessed September 15, 2026).

[33] Musotsi PY Otieno CA Njoroge SM. Prevalence of fasciolosis in cattle, sheep, and goats slaughtered in slaughter slabs in trans-Nzoia west, Kenya and knowledge of livestock handlers. J Biol Agric Healthc. (2017) 7:1–7. Available online at: http://ir.mu.ac.ke:8080/xmlui/handle/123456789/6044

[34] Opisa S Odiere MR Jura WGZO Karanja DMS Mwinzi PNM. Malacological survey and geographical distribution of vector snails for schistosomiasis within informal settlements of Kisumu City, western Kenya. Parasit Vectors. (2011) 4:226. doi: 10.1186/1756-3305-4-226, 22152486 PMC3247081

[35] Preston JM Castelino JB. A study of the epidemiology of bovine fascioliasis in Kenya and its control using N-Tritylmorpholine. Br Vet J. (1977) 133:600–8. doi: 10.1016/S0007-1935(17)33940-4, 608108

[36] Cheruiyot HK. Bovine helminth parasites of economic importance - abattoir survey in Kenya 1976-1980. Bull Anim Health Prod Afr. (1983) 31:367–75.

[37] Nginyi JM Onyango JAA Harrison LJS. Helminth and non-helminth causes of ovine and caprine liver condemnations at Kiserian abattoirs, Kenya. Bull Anim Health Prod Afr. (1995) 43:297–9.

[38] Maingi N Gichingi MN. Prevalence and economic importance of *Fasciola* and other helminth parasites of cattle and sheep in Nyandarua District of Kenya. Bull Anim Health Prod Afr. (1999) 47:29–32.

[39] Njeruh FM Kithuka JM Maingi N Ombui JN. Prevalence and economic importance of fascioliasis in cattle, sheep and goats in Kenya. Kenya Vet. (2007) 27:118–123. doi: 10.4314/kenvet.v27i1.39574

[40] Ngaira MV. The socio-economic impact of fascioliasis disease in Nandi central district, Kenya. Archiv Bus Res. (2016) 4:26–32. doi: 10.14738/abr.44.2118

[41] Mutua F Kihara A Rogena J Ngwili N Aboge G Wabacha J . Piloting a livestock identification and traceability system in the northern Tanzania-Narok-Nairobi trade route. Trop Anim Health Prod. (2018) 50:299–308. doi: 10.1007/s11250-017-1431-4, 28965304 PMC5780531

[42] Odede J. F. (2021) *Epidemiology of Bovine Fasciolosis in Kinango, Kwale County, Kenya (master's thesis)*, Kenyatta University. Kenyatta University Digital Repository

[43] Mungube E Bauni S Tenhagen B-A Wamae L Nginyi J Mugambi J. The prevalence and economic significance of *Fasciola gigantica* and *Stilesia hepatica* in slaughtered animals in the semi-arid coastal Kenya. Trop Anim Health Prod. (2006) 38:475–83. doi: 10.1007/s11250-006-4394-4, 17243475

[44] Roach RW Lopes V. Mortality in adult ewes resulting from intestinal infestation with immature paramphistomes, complicated by severe fasciolasis. Bull Epizoot Dis Afr Bull Epizoot Afr. (1966) 14:317–23. 6009234

[45] Hansen J Perry B. The Epidemiology and Control of helminth Parasites of Ruminants. 2nd ed. Nairobi, Kenya: A publication for the International Laboratory for Research on Animal Diseases (ILRAD) (1994). p. 31–43.

[46] Karanja S.M. (2005) *Epidemiology and importance of trypanosomosis, helminthosis and tickborne diseases on performance of cattle in Busia District, Kenya (PhD thesis)*, Freie Universitat Berlin

[47] Maingi N Gichohi VM Munyua WK Gathuma JM Thumsborg SM. The epidemiology of nematode and liver fluke infections in sheep in Nyandarua district of Kenya. Bull Anim Health Prod Africa. (1997) 45:27–34.

[48] Peeler EJ Wanyangu SW. Infectious causes of small ruminant mortality in Kenya: a review. Small Rumin Res. (1998) 29:1–11. doi: 10.1016/S0921-4488(97)00120-X

[49] Wamae LW Hammond JA Harrison LJS Onyango-Abuje JA. Comparison of the production losses caused by chronic *Fasciola gigantica* infection in yearling Friesian and Boran cattle. Trop Anim Health Prod. (1998) 30:23–30. doi: 10.1023/A:1005057225427, 9719825

[50] Bitakaramire PK. The survival of *Lymnaea natalensis* in drought conditions. Bull Epizoot Dis Afr. (1968b) 16:473–5. 5731207

[51] Bitakaramire PK. *Lymnaea natalensis* laboratory culture and production of *Fasciola gigantica* metacercariae. Parasitology. (1968c) 58:653–6. doi: 10.1017/S0031182000028948, 4905987

[52] Biwott G. K. Ngeiywa M. Makwali J. (2025). Abundance and distribution of snail intermediate hosts of *Fasciola* spp. a high-risk fascioliasis ecosystem in western Kenya. Ecol Evol Biol, 10, 156–167. Doi:10.11648/j.eeb.20251004.13 [1]

[53] Dida GO Gelder FB Anyona DN Matano A-S Abuom PO Adoka SO . Distribution and abundance of schistosomiasis and fascioliasis host snails along the Mara River in Kenya and Tanzania. Infect Ecol Epidemiol. (2014) 4:24281. doi: 10.3402/iee.v4.24281, 25405008 PMC4216393

[54] Wamae LW Ongare JO Ihiga MA Mahaga M. Epidemiology of fasciolosis on a ranch in the central Rift Valley, Kenya. Trop Anim Health Prod. (1990) 22:132–4. doi: 10.1007/BF02239841, 2371752

[55] Owiny MO Obonyo MO Gatongi PM Fèvre EM. Prevalence and spatial distribution of trematode cercariae in vector snails within different agro-ecological zones in Western Kenya, 2016. Pan Afr Med J. (2019) 32:142. doi: 10.11604/pamj.2019.32.142.14418, 31303914 PMC6607274

[56] Jelnes JE Ouma JH. Distribution of *Bulinus*, *Biomphalaria* and *Lymnaea* in Kenya. Trans R Soc Trop Med Hyg. (1981) 75:185–6. doi: 10.1016/0035-9203(81)90073-0

[57] Cheruiyot HK Wamae LW. Some observations on the occurence and distribution of *Radix natalensis* Krauss in its habitats in Kenya. East Afr Agric For J. (1986) 52:58–60. doi: 10.1080/00128325.1986.11663495

[58] Dinnik JA Diknik NN. The influence of temperature on the succession of redial and cercarial generations of *Fasciola gigantica* in a snail host. Parasitology. (1964) 54:59–65. doi: 10.1017/S0031182000074321, 14124283

[59] Teesdale C. Ecological observations on the molluscs of significance in the transmission of bilharziasis in Kenya. Bull World Health Organ. (1962) 27:759–82. 13980432 PMC2555847

[60] Churu H Kamau S Ngetich W Magiroi K Alkamoi B Kebeney S . Spatial–temporal influence of sand dams on chemical and microbial properties of water from scooping holes in degraded semi-arid regions. Water. (2023) 15:3207. doi: 10.3390/w15183207

[61] Gitau J.K. (2014) *Effectiveness of natural wetland in waste water treatment: a case study of Tibia Wetland, Limuru Municipality, Kenya (master's thesis)*, Kenyatta University

[62] Hisatake N Daisuke K Kenjiro K Yasuo N Jun-ichi I Makoto S . Host snails of human schistosomiasis in the Taveta area of Kenya, East Africa. Jpn J Trop Med Hyg. (1978) 6:181–93. doi: 10.2149/tmh1973.6.181

[63] Bitakaramire PK. The incidence of fascioliasis in different breeds of cattle in Kenya. Bull Epizoot Dis Afr. (1973) 21:145–52.

[64] Castelino JB Preston JM. The influence of breed and age on the prevalence of bovine fascioliasis in Kenya. Br Vet J. (1979) 135:198–203. doi: 10.1016/S0007-1935(17)32942-1, 427525

[65] Waweru JG Kanyari PW Mwangi DM Ngatia TA Nansen P. A comparison of serum biochemical changes in two breeds of sheep (red Masai and dorper) experimentally infected with *Fasciola gigantica*. Onderstepoort J Vet Res. (1999b) 66:47–9. 10396762

[66] Kagira JM Kanyari PN Githigia SM Maingi N Ng’ang’a JC Gachohi JM. Risk factors associated with occurrence of nematodes in free range pigs in Busia District, Kenya. Trop Anim Health Prod. (2012) 44:657–64. doi: 10.1007/s11250-011-9951-9, 21833678

[67] Wachira JW Shavulimo RS. (1986) Health problems of goats on Western Kenya. Proceedings of the 5th small ruminant CRSP workshop, pp. 108–112.

[68] Waruru R Mutune M Otieno R. Gastrointestinal parasite infections of sheep and goats in a semi-arid area of Machakos District, Kenya. Bull Anim Health Prod Afr. (2005) 53:25–34. doi: 10.4314/bahpa.v53i1.32686

[69] Waruiru RM Kyvsgaard NC Thamsborg SM Nansen P Bøgh HO Munyua WK . The prevalence and intensity of helminth and coccidial infections in dairy cattle in Central Kenya. Vet Res Commun. (2000) 24:39–53. doi: 10.1023/a:1006325405239, 10703753

[70] Nginyi J. M. (2002) A strategy anthelmintic control for helminthoses of sheep: some experiences in on-farm trials in smallholder farms in Central Kenya Proceedings of the 8th biennial scientific conference: demand-driven agricultural research and development for sustainable resource management and increased production (p. 74).

[71] Kuntz RE Moore JA. Intestinal parasites and commensals of an indigenous population in the Lake Baringo area of Central Kenya. Proc Helminthol Soc Wash. (1971) 38:215–7.

[72] Moll G Lohding A Young AS. Epidemiology of theilerioses in the trans-Mara division, Kenya: husbandry and disease background and preliminary investigations on theilerioses in calves. Prev Vet Med. (1984) 2:801–31. doi: 10.1016/0167-5877(84)90035-7

[73] Ngetich EC Ngeiywa M Ng’wena GM Chemwolo L. Haematological parameters by age and sex of asymptomatic indigenous cattle and sheep infected with gastrointestinal parasites in Kerio Valley, Kenya. Am J Biomed Res. (2019) 7:44–50. doi: 10.12691/ajbr-7-2-4

[74] Wafula MD. Pattern of occurrence of gastrointestinal helminthiasis in dairy goat breeds within trans Nzoia County, Kenya. South Asia J Parasitol. (2019) 2:248–54. doi: 10.9734/sajp/2019/v3i330107

[75] Hammond JA. Infections with *Fasciola* spp. in wildlife in Africa. Trop Anim Health Prod. (1972) 4:1–13. doi: 10.1007/BF02357089, 4671397

[76] King’ori E Obanda V Chiyo PI Soriguer RC Morrondo P Angelone S. Patterns of helminth infection in Kenyan elephant populations. Parasit Vectors. (2020) 13:145. doi: 10.1186/s13071-020-04017-1, 32188499 PMC7081694

[77] Kithuka JM. (2004) *The prevalence, distribution and economic importance of fasciolosis in cattle, sheep and goats in Kenya (master's thesis)*, University of Nairobi, University of Nairobi Digital Repository.

[78] Mukhwana E. (1993) *Comparison of the efficacy of three anthelmintic drugs against mixed natural gastrointestinal nematode infections in camel (Camelus dromedarius) in Kenya (master's thesis)*, University of Nairobi, University of Nairobi Digital Repository

[79] Elsheikha H Obanda V. (2010) Parasitic Impact on Elephant Conservation: A Kenyan View. Available online at: https://www.vettimes.co.uk (Accessed September 3, 2026)

[80] Rajapakse RPVJ Lawton SP Karunathilake KJK Perera BVP Nguyen NTB Le TH. Molecular characterization of *Fasciola jacksoni* from wild elephants (*Elephas maximus maximus*) of Sri Lanka: a taxonomic evaluation. Parasitology. (2019) 146:1247–55. doi: 10.1017/S0031182019000519, 31084638

[81] Waweru JG Kanyari PWN Mwangi DM Ngatia TA Nansen P. Comparative parasitological and haematological changes in two breeds of sheep infected with *Fasciola gigantica*. Trop Anim Health Prod. (1999a) 31:363–72. doi: 10.1023/A:1005221108013, 10599131

[82] Bitakaramire PK. A new technique for the recovery of *Fasciola gigantica* eggs from cattle faeces. Bull Epizoot Dis Afr. (1967) 15:389–91. 4887044

[83] Dinnik JA Dinnik NN. A method for the simultaneous diagnosis of schistosomiasis, fascioliasis and paramphistomiasis in cattle. Bull Epizoot Dis Afr Bull Epizoot Afr. (1963) 11:29–36. 5896615

[84] Kleiman F Pietrokovsky S Gil S Wisnivesky-Colli C. Comparison of two coprological methods for the veterinary diagnosis of fasciolosis. Arq Bras Med Vet Zootec. (2005) 57:181–5. doi: 10.1590/S0102-09352005000200007

[85] Moustafa NE Hegab MH Hassan MM. Role of ELISA in early detection of *Fasciola* copro-antigens in experimentally infected animals. J Egypt Soc Parasitol. (1998) 28:379–87. 9707667

[86] Broberg G Cheruiyot HK Wachira TM. Laboratory observations of the actions of *Glinus lotoides* (fam. Aizoacea) fruit on snail hosts of *Fasciola* and *Schistosoma* in Kenya. Ann Trop Med Parasitol. (1984) 78:663. doi: 10.1080/00034983.1984.11811880, 6532336

[87] Mkoji GM Njung’e K Kimani G Kofi-Tsekpo W Mungai BN Kamau T . Molluscicidal activity of *Solanum aculeatum* (family: Solanaceae) berries against *Biomphalaria pfeifferi*, *Bulinus globosus* and *Lymnaea natalensis*. Trop Med Parasitol. (1989) 40:119–20. 2772517

[88] Ogambo-Ogoma AH. The effect of clearing on the population of *Lymnaea natalensis*. Bull Epizoot Dis Afr Bull Des Epizoot En Afr. (1972) 20:25–30. 5078481

[89] Bakry FA Hamdi SAH. Effect of non target snails on some biological of *Lymnaea natalensis* snails and their infection to *Fasciola gigantica*. J Egypt Soc Parasitol. (2006) 36:979–92. 17153707

[90] Nguma JF McCullough FS Masha E. Elimination of *Biomphalaria pfeifferi*, *Bulinus tropicus* and *Lymnaea natalensis* by the ampullarid snail, *Marisa cornuarietis*, in a man-made dam in northern Tanzania. Acta Trop. (1982) 39:85–90. 6122367

[91] Dunne JA Williams RJ. Cascading extinctions and community collapse in model food webs. Phil Trans Biol Sci. (2009) 364:1711–23. doi: 10.1098/rstb.2008.0219, 19451122 PMC2685420

[92] Zhang H Sun T Cao H Zhang Y Yang W Shao D . Movement of mud snails affects population dynamics, primary production and landscape heterogeneity in tidal flat ecosystems. Landsc Ecol. (2021) 36:3493–506. doi: 10.1007/s10980-021-01322-7

[93] Maingi N Otieno RO Weda EH Gichohi VM. Effects of three anthelmintic treatment regimes against *Fasciola* and nematodes on the performance of ewes and lambs on pasture in the highlands of Kenya. Vet Res Commun. (2002) 26:543–52. doi: 10.1023/A:1020291531858, 12416869

[94] Marcos L Maco V Terashima A. Triclabendazole for the treatment of human fascioliasis and the threat of treatment failures. Expert Rev Anti-Infect Ther. (2021) 19:817–23. doi: 10.1080/14787210.2021.1858798, 33267701

[95] Hou M Gao J Liu X Zhou J Ma T Zhang Y . Triclabendazole and other fasciolicides: resistance of *Fasciola hepatica* in ruminants. Animals. (2026) 16:1044. doi: 10.3390/ani16071044, 41976023 PMC13072294

[96] Gakuya DW Mbithi PMF Maitho TE Musimba NKR. The potential use of plant anthelmintics for the control of livestock helminthoses in Kenya. Kenya Vet. (2004) 26:14–26.

[97] Githiori JB Höglund J Waller PJ Baker RL. Evaluation of anthelmintic properties of some plants used as livestock dewormers against *Haemonchus contortus* infections in sheep. Parasitology. (2004) 129:245–53. doi: 10.1017/S0031182004005566, 15376783

[98] Muthee JK Gakuya DW Mbaria JM Kareru PG Mulei CM Njonge FK. Ethnobotanical study of anthelmintic and other medicinal plants traditionally used in Loitoktok district of Kenya. J Ethnopharmacol. (2011) 135:15–21. doi: 10.1016/j.jep.2011.02.005, 21349318

[99] Thoithi G Maingi N Karume D Gathumbi P Mwangi J Kibwage I. Anthelmintic and other pharmacological activities of the root bark extracts of *Albizia anthelmintica* Brongn. East Cent Afr J Pharm Sci. (2005) 5:60–66. doi: 10.4314/ecajps.v5i3.9690

[100] Orengo KO Maitho T Mbaria JM Maingi N Kitaa JM. In vitro anthelmintic activity of *Allium sativum*, *Allium cepa* and *Jatropha curcas* against *Toxocara canis* and *Ancylostoma caninum*. Afr J Pharm Pharmacol. (2016) 10:465–71. doi: 10.5897/AJPP2016.4551

[101] Muthee J. Anthelmintic efficacy of selected medicinal plants against gastrointestinal nematodes in naturally infected sheep in Kenya. J Phytopharmacol. (2018) 7:111–5. doi: 10.31254/phyto.2018.7202

[102] Hilton M. JBI critical appraisal checklist for systematic reviews and research syntheses. J Canad Health Lib Assoc. (2024) 45:180–3. doi: 10.29173/jchla29801, 42842602

[103] Joanna Briggs Institute (2024) JBI manual for evidence synthesis (Aromataris, E., Lockwood, C., Porritt, K., Pilla, B., and Jordan, Z.Eds.). JBI. https://jbi-global-wiki.refined.site/space/MANUAL

[104] Nguyen TBN De NV Nguyen TKL Quang HH Doan HTT Agatsuma T. . (2018). Distribution Status of Hybrid Types in Large Liver Flukes, Fasciola Species (Digenea: Fasciolidae), from Ruminants and Humans in Vietnam. The Korean Journal of Parasitology, 56, 453–461. doi: 10.3347/kjp.2018.56.5.453, 30419731 PMC6243193

[105] Mahulu A Clewing C Stelbrink B Chibwana FD Tumwebaze I Russell Stothard J. . (2019). Cryptic intermediate snail host of the liver fluke Fasciola hepatica in Africa. Parasites & Vectors, 12. doi: 10.1186/s13071-019-3825-9 PMC6894237 31801595

