# Supplementary material for: Fascioliasis in Kenya: a review
Source: Front Vet Sci. 2026 Oct 26;13:1998426. doi: 10.3389/fvets.2026.1998426 (PMC13746238; doi:10.3389/fvets.2026.1998426)
Supplement: Supplementary file 1 [file Table_1.docx]

**SUPPLEMENTARY FILE 1: A list of Fascioliais relevant sources not included in the final review as they do not directly address “Fascioliasis in Kenya”**

1. Abdel‑Fatah, O. R., Arafa, W. M., Wahba, A. A., & El‑Dakhly, K. M. (2022). Economic losses, morpho-molecular identification, and identity of Fasciola species recovered from Egypt. *Journal of Parasitic Diseases*, *46*(4), 1036–1046. <https://doi.org/10.1007/s12639-022-01526-x>
2. Abd-El-Megeed, K. N. (1999). Studies on the molluscicidal actvity of *Calendula micrantha* officinalis (Compositae) on fascioliasis transmitting snails. *Journal of the Egyptian Society of Parasitology*, *29*(1), 183–192.
3. Ackley, C., Elsheikh, M., Zaman, S., & Yazdi-Feyzabadi, V. (2021). Scoping review of Neglected Tropical Disease Interventions and Health Promotion: A framework for successful NTD interventions as evidenced by the literature. *PLoS Neglected Tropical Diseases*, *15*(7). <https://doi.org/10.1371/journal.pntd.0009278>
4. Adediran, O. A., Adebiyi, A. I., & Uwalaka, E. C. (2014). Prevalence of *Fasciola* species in ruminants under extensive management system in Ibadan southwestern Nigeria. *African Journal of Medicine and Medical Sciences*, *43 Suppl*, 137–141.
5. Adewunmi, C. O., Furu, P., Marquis, B. B., Fagbola, M., & Olatunji, O. A. (1990). Molluscicidal trials and correlation between the presence of *Tetrapleura tetraptera* in an area and the absence of the intermediate hosts of schistosomiasis and fascioliasis in southwest Nigeria. *Journal of Ethnopharmacology*, *30*(2), 169–183. <https://doi.org/10.1016/0378-8741(90)90006-f>
6. Afshan, K., Fortes-Lima, C. A., Artigas, P., Valero, A. M., Qayyum, M., & Mas-Coma, S. (2014). Impact of climate change and man-made irrigation systems on the transmission risk, long-term trend and seasonality of human and animal fascioliasis in Pakistan. *Geospatial Health*, *8*(2), 317–334. <https://doi.org/10.4081/gh.2014.22>
7. Aftab, A., Lall, R., Bisen, S., Anandanarayanan, A., Rialch, A., Chamuah, J. K., Yadav, S., Silamparasan, M., & Raina, O. K. (2020). Serodiagnosis of *Fasciola gigantica* Infection in Buffaloes with Native Cathepsin-L Proteases and Recombinant Cathepsin L1-D. *Acta Parasitologica*, *65*(2), 413–421. <https://doi.org/10.2478/s11686-020-00177-3>
8. Aftab, A., Raina, O. K., Maxton, A., & Masih, S. A. (2024). Advances in diagnostic approaches to *Fasciola* infection in animals and humans: An overviews. *Journal of Helminthology*, *98*, e12. <https://doi.org/10.1017/S0022149X23000950>
9. Aghayan, S., Gevorgian, H., Ebi, D., Atoyan, H. A., Addy, F., Mackenstedt, U., Romig, T., & Wassermann, M. (2019). *Fasciola* spp. in Armenia: Genetic diversity in a global context. *Veterinary Parasitology*, *268*, 21–31. <https://doi.org/10.1016/j.vetpar.2019.02.009>
10. Ahmad, T., Imran, M., Ahmad, K., Khan, M., Baig, M., Al-Rifai, R. H., Al-Omari, B., & Mancianti, F. (2021). A Bibliometric Analysis and Global Trends in Fascioliasis Research: A Neglected Tropical Disease. *Animals : An Open Access Journal from MDPI*, *11*(12). <https://doi.org/10.3390/ani11123385>
11. Alemayehu, B., Demissie, Z., & Beyene, E. (2023). Human fascioliasis: A diagnostic challenge in resource limited setting. *Ethiopian Medical Journal*, *61*(4), 391–394. <https://doi.org/10.4314/emj.v61i4.11>
12. Alonge, D. O., & Fasanmi, E. F. (1979). A survey of abattoir data in northern Nigeria. *Tropical Animal Health and Production*, *11*(1), 57–62. <https://doi.org/10.1007/BF02237770>
13. Alvarez Rojas, C. A., Jex, A. R., Gasser, R. B., & Scheerlinck, J.-P. Y. (2014). Techniques for the diagnosis of *Fasciola* infections in animals: room for improvement. *Advances in Parasitology*, *85*, 65–107. <https://doi.org/10.1016/B978-0-12-800182-0.00002-7>
14. Amato, S. B., De Rezende, H. E., Gomes, D. C., & Da Serra Freire, N. M. (1986). Epidemiology of *Fasciola hepatica* infection in the Paraíba River Valley, São Paulo, Brasil. *Veterinary Parasitology*, *22*(3-4), 275–284.
15. Amer, S., ElKhatam, A., Zidan, S., Feng, Y., & Xiao, L. (2016). Identity of Fasciola spp. in sheep in Egypt. *Parasites & Vectors*, *9*(1), 623. <https://doi.org/10.1186/s13071-016-1898-2>
16. Angles, R., Buchon, P., Valero, M. A., Bargues, M. D., & Mas-Coma, S. (2022). One Health Action against Human Fascioliasis in the Bolivian Altiplano: Food, Water, Housing, Behavioural Traditions, Social Aspects, and Livestock Management Linked to Disease Transmission and Infection Sources. *International Journal of Environmental Research and Public Health*, *19*(3). <https://doi.org/10.3390/ijerph19031120>
17. Angles, R., Buchon, P., Valero, M. A., Bargues, M. D., & Mas-Coma, S. (2022). One Health Action against Human Fascioliasis in the Bolivian Altiplano: Food, Water, Housing, Behavioural Traditions, Social Aspects, and Livestock Management Linked to Disease Transmission and Infection Sources. *International Journal of Environmental Research and Public Health*, *19*(3). <https://doi.org/10.3390/ijerph19031120>
18. Apt, W., Aguilera, X., Vega, F., Zulantay, I., Retamal, C., Apt, P., & Sandoval, J. (1992). [Fascioliasis in populations of rural areas with high prevalence of animal infection]. *Revista Medica de Chile*, *120*(6), 621–626.
19. Arafa, W. M., Hassan, A. I., Snousi, S. A. M., El-Dakhly, K. M., Holman, P. J., Craig, T. M., & Aboelhadid, S. M. (2018). Fasciola hepatica infections in cattle and the freshwater snail *Galba truncatula* from Dakhla Oasis, Egypt. *Journal of Helminthology*, *92*(1), 56–63. <https://doi.org/10.1017/S0022149X17000086>
20. Archimandritis, A., Theodoropoulos, G., Bartsokas, S., & Melissinos, K. (1976). Liver fascioliasis, a “mysterious” disease. Report of a case. *Acta Hepato-Gastroenterologica*, *23*(6), 409–411.
21. Arias-Pacheco, C., Lucas, J. R., Rodríguez, A., Córdoba, D., & Lux-Hoppe, E. G. (2020). Economic impact of the liver condemnation of cattle infected with Fasciola hepatica in the Peruvian Andes. *Tropical Animal Health and Production*, *52*(4), 1927–1932. <https://doi.org/10.1007/s11250-020-02211-y>
22. Arjona, R., Riancho, J. A., Aguado, J. M., Salesa, R., & González-Macías, J. (1995). Fascioliasis in developed countries: a review of classic and aberrant forms of the disease. *Medicine*, *74*(1), 13–23. <https://doi.org/10.1097/00005792-199501000-00002>
23. Armour, J., & Urquhart, G. M. (1974). The Control of Helminthiasis in Ruminants. *British Veterinary Journal*, *130*(2), 99–109. <https://doi.org/10.1016/S0007-1935(17)35932-8>
24. Ashrafi, K. (2015). The Status of Human and Animal Fascioliasis in Iran: A Narrative Review Article. *Iranian Journal of Parasitology*, *10*(3), 306–328
25. Ashrafi, K., Bargues, M. D., O’Neill, S., & Mas-Coma, S. (2014). Fascioliasis: A worldwide parasitic disease of importance in travel medicine. *Travel Medicine and Infectious Disease*, *12*(6), 636–649. <https://doi.org/10.1016/j.tmaid.2014.09.006>
26. Ashrafi, K., Saadat, F., O'Neill, S. M., Rahmati, B., Tahmasbi, H. A., Dalton, J. P., Nadim, A., Asadinezhad, M., & Rezvani, S. M. (2015). The Endemicity of Human Fascioliasis in Guilan Province, Northern Iran: the Baseline for Implementation of Control Strategies. *Iranian Journal of Public Health*, *44*(4).
27. Ashton, C. R., & Beresford, O. D. (1974). Letter: Fascioliasis. *British Medical Journal*, *2*(5910), 121. <https://doi.org/10.1136/bmj.2.5910.121-b>
28. Atalay, F., Kirimlioğlu, V., Dağli, U., Akincioğlu, T., Akoğlu, M., & Seven, C. (1993). Human fascioliasis. *Surgery Today*, *23*(4), 366–369. <https://doi.org/10.1007/BF00309057>
29. Atanga, H. M., Ntonifor, N. H., & Mahamat, O. (2024). Prevalence of bovine fasciolosis and direct financial losses in cattle slaughtered in Bamenda and Bafoussam abattoirs, Western Highlands, Cameroon. *Veterinary Medicine and Science*, *10*(6), e70002. <https://doi.org/10.1002/vms3.70002>
30. Azimov, S. A., Salimov, B. S., Nazarov, A. N., & Ernazarov, D. (1968). [Controlling fascioliasis in ruminants]. *Veterinariia*, *45*(2), 65–66.
31. Babianskas, M., Vishniauskas, A., Burakauskas, A., & Svabonas, Z. (1967). [Coprological diagnosis of fascioliasis]. *Veterinariia*, *44*(6), 58–59.
32. Bacq, Y., Besnier, J. M., Duong, T. H., Pavie, G., Metman, E. H., & Choutet, P. (1991). Successful treatment of acute fascioliasis with bithionol. *Hepatology (Baltimore, Md.)*, *14*(6), 1066–1069.
33. Bakhshipour, F., Zibaei, M., Rokni, M. B., Miahipour, A., Firoozeh, F., Beheshti, M., Beikzadeh, L., Alizadeh, G., Aryaeipour, M., & Raissi, V. (2024). Comparative evaluation of real-time PCR and ELISA for the detection of human fascioliasis. *Scientific Reports*, *14*(1), 3865. <https://doi.org/10.1038/s41598-024-54602-y>
34. Baldock, F. C., & Arthur, R. J. (1985). A survey of fascioliasis in beef cattle killed at abattoirs in southern Queensland. *Australian Veterinary Journal*, *62*(10), 324–326. <https://doi.org/10.1111/j.1751-0813.1985.tb07650.x>
35. Barcat, J. A. (2005). [Watercress and other dangerous foods]. *Medicina*, *65*(3), 277–279.
36. Barduagni, P., Hassanein, Y., Mohamed, M., Wakeel, A. E., Sayed, M. E., Hallaj, Z., & Curtale, F. (2008). Use of triclabendazole for treatment of patients co-infected by *Fasciola* spp. and *S. mansoni* in Behera Governorate, Egypt. *Parasitology Research*, *102*(4), 631–633. <https://doi.org/10.1007/s00436-007-0802-6>
37. Bargues, M. D., & Mas-Coma, S. (2005). Reviewing lymnaeid vectors of fascioliasis by ribosomal DNA sequence analyses. *Journal of Helminthology*, *79*(3), 257–267. <https://doi.org/10.1079/joh2005297>
38. Bassily, S., Iskander, M., Youssef, F. G., el-Masry, N., & Bawden, M. (1989). Sonography in diagnosis of fascioliasis. *Lancet (London, England)*, *1*(8649), 1270–1271. <https://doi.org/10.1016/s0140-6736(89)92368-4>
39. Beesley, N. J., Caminade, C., Charlier, J., Flynn, R. J., Hodgkinson, J. E., Martinez-Moreno, A., Martinez-Valladares, M., Perez, J., Rinaldi, L., & Williams, D. J. L. (2018). *Fasciola* and fasciolosis in ruminants in Europe: Identifying research needs. *Transboundary and Emerging Diseases*, *65*, 199–216. <https://doi.org/10.1111/tbed.12682>
40. Bennema, S. C., Ducheyne, E., Vercruysse, J., Claerebout, E., Hendrickx, G., & Charlier, J. (2011). Relative importance of management, meteorological and environmental factors in the spatial distribution of *Fasciola hepatica* in dairy cattle in a temperate climate zone. *International Journal for Parasitology*, *41*(2), 225–233. <https://doi.org/10.1016/j.ijpara.2010.09.003>
41. Bennett, R. M., Christiansen, K., & Clifton-Hadley, R. S. (1999). Estimating the costs associated with endemic diseases of dairy cattle. *Journal of Dairy Research*, *66*(3), 455–459. <https://doi.org/10.1017/S0022029999003684>
42. Bergquist, R., Brattig, N. W., Chimbari, M. J., Zinsstag, J., & Utzinger, J. (2017). Ecohealth research in Africa: Where from—Where to? *Acta Tropica*, *175*, 1–8. <https://doi.org/10.1016/j.actatropica.2017.07.015>
43. Black, J., Ntusi, N., Stead, P., Mayosi, B., & Mendelson, M. (2013). Human fascioliasis in South Africa. *South African Medical Journal = Suid-Afrikaanse Tydskrif Vir Geneeskunde*, *103*(9), 658–659. <https://doi.org/10.7196/samj.7184>
44. Bogan, J., & Armour, J. (1987). Anthelmintics for ruminants. *International Journal for Parasitology*, *17*(2), 483–491. <https://doi.org/10.1016/0020-7519(87)90124->
45. Boray, J. C., & Happich, F. A. (1968). Standardised chemotherapeutical tests for immature and mature *Fasciola hepatica* infections in sheep. *Australian Veterinary Journal*, *44*(2), 72–78. <https://doi.org/10.1111/j.1751-0813.1968.tb04960.x>
46. Boray, J. C., Fraser, G. C., Williams, J. D., & Wilson, J. M. (1985). The occurrence of the snail *Lymnaea columella* on grazing areas in New South Wales and studies on its susceptibility to *Fasciola hepatica*. *Australian Veterinary Journal*, *62*(1), 4–6. <https://doi.org/10.1111/j.1751-0813.1985.tb06030.x>
47. Boray, J., Jackson, R., & Strong, M. (1985). Chemoprophylaxis of fascioliasis with triclabendazole. *New Zealand Veterinary Journal*, *33*(11), 182–185.
48. Bosco, A., Ciuca, L., Maurelli, M. P., Vitiello, P., Cringoli, G., Prada, J. M., & Rinaldi, L. (2023). Comparison of Mini-FLOTAC, Flukefinder® and sedimentation techniques for detection and quantification of *Fasciola* *hepatica* and *Calicophoron daubneyi* eggs using spiked and naturally infected bovine faecal samples. *Parasites & Vectors*, *16*(1). <https://doi.org/10.1186/s13071-023-05890-2>
49. Bossche, H.V. (1985). How anthelmintics help us to understand helminths. *Parasitology*, *90 ( Pt 4)*, 675–685. <https://doi.org/10.1017/s0031182000052306>
50. Boulliat, C., Wolf, A., Gaillard, K., & Oliver, M. (2010). [Triclabendazole]. *Medecine Tropicale : Revue Du Corps de Sante Colonial*, *70*(4), 341–342.
51. Braun, F. (1968). [Contributions to the fight against Galba truncatula and Fasciola hepatica]. *Zeitschrift Fur Parasitenkunde (Berlin, Germany)*, *31*(1), 15–16. <https://doi.org/10.1007/BF00716417>
52. Bruce, R. G., Armour, J., & Corba, J. (1973). A further study of the epidemiology of ovine fascioliasis in Scotland and its control using molluscicide. *The Veterinary Record*, *92*(20), 518–526. <https://doi.org/10.1136/vr.92.20.518>
53. Bryan, F. L. (1977). Diseases Transmitted by Foods Contaminated by Wastewater. *Journal of Food Protection*, *40*(1), 45–56. <https://doi.org/10.4315/0362-028X-40.1.45>
54. Buckner, A. (2021). Prevalence and management of liver fluke in the UK. *The Veterinary Record*, *188*(3), 115. <https://doi.org/10.1002/vetr.174>
55. Buckner, A. (2021). Prevalence and management of liver fluke in the UK. *The Veterinary Record*, *188*(3), 115. <https://doi.org/10.1002/vetr.174>
56. Cabada, M. M., & White, A. C. (2012). New developments in epidemiology, diagnosis, and treatment of fascioliasis. *Current Opinion in Infectious Diseases*, *25*(5), 518–522. <https://doi.org/10.1097/QCO.0b013e3283567b7e>
57. Cabada, M. M., Malaga, J. L., Castellanos-Gonzalez, A., Bagwell, K. A., Naeger, P. A., Rogers, H. K., Maharsi, S., Mbaka, M., & White, A. C. (2017). Recombinase Polymerase Amplification Compared to Real-Time Polymerase Chain Reaction Test for the Detection of Fasciola hepatica in Human Stool. *The American Journal of Tropical Medicine and Hygiene*, *96*(2), 341–346. <https://doi.org/10.4269/ajtmh.16-0601>
58. Cadmus, S. I. B., & Adesokan, H. K. (2009). Causes and implications of bovine organs/offal condemnations in some abattoirs in Western Nigeria. *Tropical Animal Health and Production*, *41*(7), 1455–1463. <https://doi.org/10.1007/s11250-009-9334-7>
59. Caminal Montero, L., Fernández Fernández, C., de Quirós, J. F., & Parra, F. (1999). [The treatment of human fascioliasis with triclabendazole]. *Revista Clinica Espanola*, *199*(5), 333–335.
60. Caravedo MA, & Cabada MM. (2020). Human Fascioliasis: Current Epidemiological Status and Strategies for Diagnosis, Treatment, and Control. *Research and Reports in Tropical Medicine*, *11*, 149–158.
61. Carmona, C., & Tort, J. F. (2017). Fasciolosis in South America: epidemiology and control challenges. *Journal of Helminthology*, *91*(2), 99–109. https://doi.org/10.1017/S0022149X16000560
62. Carnevale, S., Pantano, M. L., Kamenetzky, L., Malandrini, J. B., Soria, C. C., & Velásquez, J. N. (2015). Molecular diagnosis of natural fasciolosis by DNA detection in sheep faeces. *Acta Parasitologica*, *60*(2), 211–217. <https://doi.org/10.1515/ap-2015-0030>
63. Carnevale, S., Rodríguez, M. I., Santillán, G., Labbé, J. H., Cabrera, M. G., Bellegarde, E. J., Velásquez, J. N., Trgovcic, J. E., & Guarnera, E. A. (2001). Immunodiagnosis of human fascioliasis by an enzyme-linked immunosorbent assay (ELISA) and a micro-ELISA. *Clinical and Diagnostic Laboratory Immunology*, *8*(1), 174–177. <https://doi.org/10.1128/CDLI.8.1.174-177.2001>
64. Carrada-Bravo, T. (2003). [Fascioliasis: diagnosis, epidemiology and treatment]. *Revista de Gastroenterologia de Mexico*, *68*(2), 135–142.
65. Castilla Gómez de Agüero, V., Luka, J., Gandasegui, J., Valderas-García, E., Ajanusi, O. J., Chiezey, N. P., & Martínez-Valladares, M. (2020). *Fasciola hepatica* and *Fasciola gigantica* coexistence in domestic ruminants in Nigeria: application of a PCR-based tool. *Tropical Animal Health and Production*, *52*(6), 3893–3897. <https://doi.org/10.1007/s11250-020-02333-3>
66. Castro-Hermida, J. A., González-Warleta, M., Martínez-Sernández, V., Ubeira, F. M., & Mezo, M. (2021). Current Challenges for Fasciolicide Treatment in Ruminant Livestock. *Trends in Parasitology*, *37*(5), 430–444. <https://doi.org/10.1016/j.pt.2020.12.003>
67. Cawdery, M. J., Strickland, K. L., Conway, A., & Crowe, P. J. (1977). Production effects of liver fluke in cattle. I. The effects of infection on liveweight gain, feed intake and food conversion efficienty in beef cattle. *The British Veterinary Journal*, *133*(2), 145–159. <https://doi.org/10.1016/s0007-1935(17)34136-2>
68. Chai, J.-Y., & Jung, B.-K. (2022). General overview of the current status of human foodborne trematodiasis. *Parasitology*, *149*(10), 1262–1285. <https://doi.org/10.1017/S0031182022000725>
69. Chakroborty, A., Dankwa, B., Crusco, A., Forde-Thomas, J. E., Abeysekara, S., Kiwanuka, Z., Ley, M., Davey, S. D., Hollinshead, J., Bartholomew, B., Wild, C., Fenn, C., Thomas, D. A., Holdsworth, P., Fisher, M., Baird, M. S., Nash, R. J., & Hoffmann, K. F. (2025). Anthelmintic evaluation of triterpenes derived from conifers (family Pinaceae) on the digenean parasite Fasciola hepatica. *Fitoterapia*, *185*. <https://doi.org/10.1016/j.fitote.2025.106762>
70. Chand, M. A., Herman, J. S., Partridge, D. G., Hewitt, K., & Chiodini, P. L. (2009). Imported human fascioliasis, United Kingdom. *Emerging Infectious Diseases*, *15*(11), 1876–1877. <https://doi.org/10.3201/eid1511.090511>
71. Charlier, J., De Meulemeester, L., Claerebout, E., Williams, D., & Vercruysse, J. (2008). Qualitative and quantitative evaluation of coprological and serological techniques for the diagnosis of fasciolosis in cattle. *Veterinary Parasitology*, *153*(1-2), 44–51. <https://doi.org/10.1016/j.vetpar.2008.01.035>
72. Charlier, J., Hostens, M., Jacobs, J., Van Ranst, B., Duchateau, L., & Vercruysse, J. (2012). Integrating fasciolosis control in the dry cow management: the effect of closantel treatment on milk production. *PloS One*, *7*(8), e43216. <https://doi.org/10.1371/journal.pone.0043216>
73. Charlier, J., Vercruysse, J., Morgan, E., Van Dijk, J., & Williams, D. J. L. (2014). Recent advances in the diagnosis, impact on production and prediction of Fasciola hepatica in cattle. *Parasitology*, *141*(3), 326–335. <https://doi.org/10.1017/S0031182013001662>
74. Chen, M. X., Zhang, R. L., Xu, X. N., Yu, Q., Huang, D. N., Liu, W., Chen, S. H., Song, P., Lu, L., Cai, Y. C., Ai, L., & Chen, J. X. (2020). Parasitological and molecular detection of human fascioliasis in a young man from Guizhou, China. *Tropical Biomedicine*, *37*(1), 50–57.
75. Chevis, R., & Kelly, J. (1978). The anthelmintic activity of some benzimidazole derivatives against Fasciola hepatica. *New Zealand Veterinary Journal*, *26*(9), 218–220.
76. Chikowore, T. J., Zishiri, O. T., & Mukaratirwa, S. (2019). Phylogenetic analysis of *Fasciola* spp. isolated from slaughtered cattle in KwaZulu-Natal and Mpumalanga provinces of South Africa based on the cytochrome c oxidase subunit I mitochondrial marker. *The Onderstepoort Journal of Veterinary Research*, *86*(1), e1–e11. https://doi.org/10.4102/ojvr.v86i1.1706
77. Clark, B. M., Lloyd, B. A., Christopher, G. W., & Foody, W. F. (2005). A young man from Peru with fever and abdominal pain. *Clinical Infectious Diseases : An Official Publication of the Infectious Diseases Society of America*, *40*(6), 842–843, 879–880. <https://doi.org/10.1086/428126>
78. Clarke, V. D., Shiff, C. J., & Blair, D. M. (1961). The control of snail hosts of bilharziasis and fascioliasis in Southern Rhodesia. *Bulletin of the World Health Organization*, *25*, 549–558.
79. Claxton, J. R., Zambrano, H., Ortiz, P., Delgado, E., Escurra, E., & Clarkson, M. J. (1998). Strategic control of fasciolosis in the inter-Andean valley of Cajamarca, Peru. *The Veterinary Record*, *143*(2), 42–45. <https://doi.org/10.1136/vr.143.2.42>
80. Coles, G. C. (2006). Treatment of fascioliasis in human infections. *Transactions of the Royal Society of Tropical Medicine and Hygiene*, *100*(2), 187; author reply 187–8. <https://doi.org/10.1016/j.trstmh.2005.08.002>
81. Combes, C., & Cheng, T. C. (1986). Control of biomedically important molluscs. *Archives de l’Institut Pasteur d’Algerie. Institut Pasteur d’Algerie*, *55*, 153–193.
82. Conceição, M. A. P., Durão, R. M., Costa, I. H., & da Costa, J. M. C. (2002). Evaluation of a simple sedimentation method (modified McMaster) for diagnosis of bovine fascioliosis. *Veterinary Parasitology*, *105*(4), 337–343. <https://doi.org/10.1016/s0304-4017(02)00016-x>
83. Corrales, Y., Ferrer, E., Fernández, J., Gauta, J., García, M., Aguirre, A., & Pérez, A. (2021). Diagnosis and Risk Factors of Bovine and Human Fascioliasis in Cattle Farms from a Venezuelan Andean Rural Area. *Acta Parasitologica*, *66*(3), 1048–1054. <https://doi.org/10.1007/s11686-021-00341-3>
84. Correa, A. C., Escobar, J. S., Durand, P., Renaud, F., David, P., Jarne, P., Pointier, J.-P., & Hurtrez-Boussès, S. (2010). Bridging gaps in the molecular phylogeny of the Lymnaeidae (Gastropoda: Pulmonata), vectors of Fascioliasis. *BMC Evolutionary Biology*, *10*, 381. <https://doi.org/10.1186/1471-2148-10-381>
85. Cringoli, G., Rinaldi, L., Veneziano, V., & Genchi, C. (2006). A dual anthelmintic treatment strategic scheme for the control of fasciolosis in dairy sheep farms. *Parasitology Research*, *99*(6), 700–705. <https://doi.org/10.1007/s00436-006-0223-y>
86. Croese, J., Chapman, G., & Gallagher, N. D. (1982). Evolution of Fascioliasis after Eating Wild Watercress. *Australian and New Zealand Journal of Medicine*, *12*(5), 525–527. <https://doi.org/10.1111/j.1445-5994.1982.tb03837.x>
87. Crossland, N. O. (1976). The effect of the molluscicide N-tritylmorpholine on transmission of Fasciola hepatica. *The Veterinary Record*, *98*(3), 45–48. <https://doi.org/10.1136/vr.98.3.45>
88. Cuervo, P. F., Mera Y Sierra, R., Artigas, P., Fantozzi, M. C., Bargues, M. D., & Mas-Coma, S. (2025). Impact of climate change on the spread of fascioliasis into the extreme south of South America. *PLoS Neglected Tropical Diseases*, *19*(8), e0013433. <https://doi.org/10.1371/journal.pntd.0013433>
89. Curtale, F. (2008). Treatment of human fascioliasis with triclabendazole: good news. *Transactions of the Royal Society of Tropical Medicine and Hygiene*, *102*(5), 508. <https://doi.org/10.1016/j.trstmh.2008.02.002>
90. Curtale, F., Abd El-Wahab Hassanein, Y., El Wakeel, A., Mas-Coma, S., & Montresore, A. (2003). Distribution of human fascioliasis by age and gender among rural population in the Nile Delta, Egypt. *Journal of Tropical Pediatrics*, *49*(5), 264–268. <https://doi.org/10.1093/tropej/49.5.264>
91. Curtale, F., Hassanein, Y. A. E. W., & Savioli, L. (2005). Control of human fascioliasis by selective chemotherapy: Design, cost and effect of the first public health, school-based intervention implemented in endemic areas of the Nile Delta, Egypt. *Transactions of the Royal Society of Tropical Medicine and Hygiene*, *99*(8), 599–609. <https://doi.org/10.1016/j.trstmh.2005.03.004>
92. Curtale, F., Mas-Coma, S., Hassanein, Y. A. E. W., Barduagni, P., Pezzotti, P., & Savioli, L. (2003). Clinical signs and household characteristics associated with human fascioliasis among rural population in Egypt: a case-control study. *Parassitologia*, *45*(1), 5–11.
93. Cwiklinski, K., O’Neill, S. M., Donnelly, S., & Dalton, J. P. (2016). A prospective view of animal and human Fasciolosis. *Parasite Immunology*, *38*(9), 558–568. <https://doi.org/10.1111/pim.12343>
94. Dahesa, G. D., Kebede, I. A., Tilahun, A. A., Borena, B. M., Muleta, Y. J., Gebremeskel, H. F., Wadajo, S. R., Lemu, G. H., & Gudina, S. B. (2026). Co-infection of fasciolosis and hydatidosis in cattle at Jimma Municipal Abattoir, Ethiopia: Prevalence, risk factors, and economic impact. *Parasitology Research*, *125*(1). <https://doi.org/10.1007/s00436-026-08666-6>
95. Dar, Y. D., Rondelaud, D., & Dreyfuss, G. (2005). Update of fasciolosis-transmitting snails in Egypt (review and comment). *Journal of the Egyptian Society of Parasitology*, *35*(2), 477–490.
96. Dar, Y., Djuikwo, T. F. F., Vignoles, P., Dreyfuss, G., & Rondelaud, D. (2010). Radix natalensis (Gastropoda: Lymnaeidae), a potential intermediate host of Fasciola hepatica in Egypt. *Parasite (Paris, France)*, *17*(3), 251–256. <https://doi.org/10.1051/parasite/2010173251>
97. Dar, Y., Rondelaud, D., & Dreyfuss, G. (2003). Cercarial shedding from *Galba* *truncatula* infected with *Fasciola gigantica* of distinct geographic origins. *Parasitology Research*, *89*(3), 185–187. <https://doi.org/10.1007/s00436-002-0732-2>
98. Dauchy, F. A., Vincendeau, P., & Lifermann, F. (2006). Eight cases of fascioliasis: clinical and microbiological features. *Medecine et Maladies Infectieuses*, *36*(1), 42–46. <https://doi.org/10.1016/j.medmal.2005.10.005>
99. Dawes, B., & Hughes, D. L. (1964). Fascioliasis: the Invasive Stages of *Fasciola hepatica* in Mammalian Hosts. In *Advances in Parasitology* (pp. 97–168). <https://doi.org/10.1016/S0065-308X(08)60587-4>
100. de Almeida, T. M., de Oliveira, F., das Chagas Xavier, S. C., Alda, P., & Molento, M. B. (2025). Ecological niche and distribution patterns of snails that host Fasciola hepatica across South America: Unveiling the hidden landscapes. *Veterinary Parasitology*, *338*. <https://doi.org/10.1016/j.vetpar.2025.110541>
101. de Boorder, J. (1992). Spread of F gigantica in Tanzania. *The Veterinary Record*, *131*(8), 180.
102. de Kock, K. N., Wolmarans, C. T., & Bornman, M. (2003). Distribution and habitats of the snail *Lymnaea truncatula*, intermediate host of the liver fluke Fasciola hepatica, in South Africa. *Journal of the South African Veterinary Association*, *74*(4), 117–122. <https://doi.org/10.4102/jsava.v74i4.523>
103. Diaw, O. T., Seye, M. M., Seye, M., Sarr, Y., & Vassiliadès, G. (1994). [Immunodiagnosis of *Fasciola gigantica* fascioliasis by ELISA technique in Senegal. Preliminary observations in 2 lambs]. *Revue d’Elevage et de Medecine Veterinaire Des Pays Tropicaux*, *47*(3), 291–294.
104. Diyana, J. N. A., Lokman, I. H., Fazila, S. H. N., Latiffah, H., Ibitoye, E. B., Hazfalinda, H. N., Chandrawathani, P., Juriah, K., & Mahiza, M. I. N. (2019). A Retrospective Study on Bovine Fascioliasis in Veterinary Regional Laboratories in Peninsular Malaysia. *Journal of Parasitology Research*, *2019*, 7903682. <https://doi.org/10.1155/2019/7903682>
105. Dow, C., Ross, J. G., & Todd, J. R. (1967). The pathology of experimental fascioliasis in calves. *Journal of Comparative Pathology*, *77*(4), 377, IN1, 385–384, IN3, 385. <https://doi.org/10.1016/0021-9975(67)90022-9>
106. Dowling, A., Lawrence, K. E., Howe, L., Scott, I., & Pomroy, W. (2025). The milk production impacts of liver fluke (*Fasciola hepatica*) infection in dairy cows on the West Coast of the South Island, New Zealand. *Veterinary Parasitology: Regional Studies and Reports*, *60*. https://doi.org/10.1016/j.vprsr.2025.101239
107. Dreyfuss, G., Vignoles, P., Abrous, M., & Rondelaud, D. (2002). Unusual snail species involved in the transmission of *Fasciola hepatica* in watercress beds in central France. *Parasite (Paris, France)*, *9*(2), 113–120. <https://doi.org/10.1051/parasite/2002092113>
108. Dronamraju, S. S., Jaques, B. C., Schmid, M., Samuel, J. R., & Charnley, R. M. (2014). Fascioliasis: a rare cause of liver abscess. *Postgraduate Medical Journal*, *90*(1061), 179–180. https://doi.org/10.1136/postgradmedj-2013-131942
109. Dugernier, T., Geubel, A., Bigaignon, G., Cesbron, J. Y., & Coche, E. (1986). Human fascioliasis: cure by mebendazole? A case report. *Gastroenterologie Clinique et Biologique*, *10*(6-7), 513–516.
110. el Shazly, A. M., Handousa, A. E., Youssef, M. E., Rizk, H., & Hamouda, M. M. (1991). Human fascioliasis: a parasitic health problem in Dakahlia Governorate, Egypt. *Journal of the Egyptian Society of Parasitology*, *21*(2), 553–559.
111. el-Aziz, M. M., Ghazy, A. A., & Effat, M. M. (2001). Immunodiagnosis of bovine fasciolosis using *Fasciola hepatica* excretory-secretory antigens ELISA. *Journal of the Egyptian Society of Parasitology*, *31*(1), 327–334.
112. el-Din, A. T. S. (2006). Molluscicidal effect of three monoterpenes oils on schistosomiasis and fascioliasis vector snails in Egypt. *Journal of the Egyptian Society of Parasitology*, *36*(2), 599–612.
113. Elelu, N., & Eisler, M. C. (2018). A review of bovine fasciolosis and other trematode infections in Nigeria. *Journal of Helminthology*, *92*(2), 128–141. <https://doi.org/10.1017/S0022149X17000402>
114. el-Karaksy, H., Hassanein, B., Okasha, S., Behairy, B., & Gadallah, I. (1999). Human fascioliasis in Egyptian children: successful treatment with triclabendazole. *Journal of Tropical Pediatrics*, *45*(3), 135–138. <https://doi.org/10.1093/tropej/45.3.135>
115. el-Morshedy, H., Farghaly, A., Sharaf, S., Abou-Basha, L., & Barakat, R. (1999). Triclabendazole in the treatment of human fascioliasis: a community-based study. *Eastern Mediterranean Health Journal = La Revue de Sante de La Mediterranee Orientale = Al-Majallah Al-Sihhiyah Li-Sharq Al-Mutawassit*, *5*(5), 888–894.
116. el-Sayed, K. A. (2006). Molluscicidal activities of aqueous extract of the sea anemone Parasicyonis actinostoloides against vector snails Bulinus truncatus and Lymnaea natalensis. *Journal of the Egyptian Society of Parasitology*, *36*(2), 531–543.
117. el-Shabrawi, M., el-Karaksy, H., Okasha, S., & el-Hennawy, A. (1997). Human fascioliasis: clinical features and diagnostic difficulties in Egyptian children. *Journal of Tropical Pediatrics*, *43*(3), 162–166. <https://doi.org/10.1093/tropej/43.3.162>
118. El-Tahawy, A. S., Kwan, N., & Sugiura, K. (2018). Fasciola hepatica infection in water buffalo Bubalus bubalis in three provinces of the Nile Delta, Egypt: a cross-sectional study. *The Journal of Veterinary Medical Science*, *80*(1), 28–35. <https://doi.org/10.1292/jvms.17-0282>
119. Eriksen, E., Eriksen, L., & Nansen, P. (1973). [Outbreak of fascioliasis (F. hepatica) in zoo animals. A clinical report]. *Nordisk Veterinaermedicin*, *25*(10), 528–533.
120. Espino, A. M., B2rges, A., & Duménigo, B. E. (2000). [Fecal antigens of Fasciola hepatica potentially useful in the diagnosis of fascioliasis]. *Revista Panamericana de Salud Publica = Pan American Journal of Public Health*, *7*(4), 225–231. <https://doi.org/10.1590/s1020-49892000000400003>
121. Estuningsih, S. E., Stevenson, P., Beriajaya, & Knox, M. R. (1990). Triclabendazole in the treatment of *Fasciola gigantica* infection in swamp buffalo (Bubalus bubalis). *Australian Veterinary Journal*, *67*(6), 234–235. <https://doi.org/10.1111/j.1751-0813.1990.tb07774.x>
122. Farag, H. F., Barakat, R. M., Ragab, M., & Omar, E. (1979). A focus of human fascioliasis in the Nile Delta, Egypt. *The Journal of Tropical Medicine and Hygiene*, *82*(9-10), 188–190.
123. Farid, Z., Mansour, N., Kamal, M., Kamal, K., Safwat, Y., & Woody, J. N. (1990). The treatment of acute *Fasciola hepatica* infection in children. *Tropical and Geographical Medicine*, *42*(1), 95–96.
124. Farrell, C. J., Shen, D. T., Wescott, R. B., & Lang, B. Z. (1981). An enzyme-linked immunosorbent assay for diagnosis of *Fasciola hepatica* infection in cattle. *American Journal of Veterinary Research*, *42*(2), 237–240.
125. Fawzy, R. K., Salem, A. E., & Osman, M. M. (1992). Ultrasonographic findings in the gall bladder in human fascioliasis. *Journal of the Egyptian Society of Parasitology*, *22*(3), 827–831.
126. Fennouh, C., Mokrani, D., Dahmani, H., Salhi, O., Ouchetati, I., Nabi, M., Aouina, K., Chettih, I., Ouchene, N., & Khelifi Touhami, N. A. (2025). Fasciolosis in Algerian livestock: Epidemiological trends and economic consequences. *Journal of Helminthology*, *99*, e77. <https://doi.org/10.1017/S0022149X25100503>
127. Fiss, L., de Lourdes Adrien, M., Marcolongo-Pereira, C., Assis-Brasil, N. D., Sallis, E. S. V., Riet-Correa, F., Ruas, J. L., & Schild, A. L. (2013). Subacute and acute fasciolosis in sheep in southern Brazil. *Parasitology Research : Founded as Zeitschrift Für Parasitenkunde*, *112*(2), 883–887. https://doi.org/10.1007/s00436-012-3096-2
128. Fraga de Azevedo, J., & Rombert, P. C. (1965). [The application of immunofluorescence to the diagnosis of hepatic fascioliasis]. *Annales de Parasitologie Humaine et Comparee*, *40*(5), 529–542.
129. Gadzhiev, I. G. (1975). [Experience with controlling fascioliasis]. *Veterinariia*, *3*, 70–71.
130. Garcia, H. H., Moro, P. L., & Schantz, P. M. (2007). Zoonotic helminth infections of humans: echinococcosis, cysticercosis and fascioliasis. *Current Opinion in Infectious Diseases*, *20*(5), 489–494. <https://doi.org/10.1097/QCO.0b013e3282a95e39>
131. Getachew, M., Innocent, G. T., Trawford, A. F., Reid, S. W. J., & Love, S. (2010). Epidemiological features of fasciolosis in working donkeys in Ethiopia. *Veterinary Parasitology*, *169*(3-4), 335–339. <https://doi.org/10.1016/j.vetpar.2010.01.007>
132. Gohar, H. A., & El-Gindy, H. I. (1961). Food of snail vectors of bilharziasis and fascioliasis. *The Journal of the Egyptian Medical Association*, *44*, 976–982.
133. Good, R., & Scherbak, D. (2023). Fascioliasis. In StatPearls. StatPearls Publishing.
134. Gorman, T., Aballay, J., Fredes, F., Silva, M., Aguillón, J. C., & Alcaíno, H. A. (1997). Immunodiagnosis of fasciolosis in horses and pigs using western blots. *International Journal for Parasitology*, *27*(11), 1429–1432. <https://doi.org/10.1016/s0020-7519(97)00090-8>
135. Graham, C. S., Brodie, S. B., & Weller, P. F. (2001). Imported Fasciola hepatica infection in the United States and treatment with triclabendazole. *Clinical Infectious Diseases: An Official Publication of the Infectious Diseases Society of America*, *33*(1), 1–5. <https://doi.org/10.1086/320870>
136. Grelck, H., & Hörchner, F. (1977). [Comparative studies on the serodiagnosis of bovine fasciolosis using the indirect immunofluorescence and immunoperoxidase test (ELISA)]. *Berliner Und Munchener Tierarztliche Wochenschrift*, *90*(17), 332–335.
137. Greter, H., Cowan, N., Ngandolo, B. N., Kessely, H., Alfaroukh, I. O., Utzinger, J., Keiser, J., & Zinsstag, J. (2017). Treatment of human and livestock helminth infections in a mobile pastoralist setting at Lake Chad: Attitudes to health and analysis of active pharmaceutical ingredients of locally available anthelminthic drugs. *Acta Tropica*, *175*, 91–99. <https://doi.org/10.1016/j.actatropica.2016.05.012>
138. Grotra, R., Malik, R., Varadarajan, A., Kandasamy, D., Yadav, R., & Gupta, S. (2025). Human Fascioliasis a Silent Menace: Case Report With Review of Literature. *Journal of Clinical and Experimental Hepatology*, *15*(3). <https://doi.org/10.1016/j.jceh.2024.102493>
139. Guobadia, E. E., & Fagbemi, B. O. (1997). The isolation of *Fasciola* *gigantica*-specific antigens and their use in the serodiagnosis of fasciolosis in sheep by the detection of circulating antigens. *Veterinary Parasitology*, *68*(3), 269–282. <https://doi.org/10.1016/s0304-4017(96)01065-5>
140. Hadden, J. W., & Pascarelli, E. F. (1967). Diagnosis and Treatment of Human Fascioliasis. *JAMA: The Journal of the American Medical Association*, *202*(2), 149–151. <https://doi.org/10.1001/jama.1967.03130150117030>
141. Hadebe, M. I., Manyangadze, T., Kalinda, C., & Chimbari, M. J. (2025). Factors contributing to the abundance and spatial distribution of the invasive intermediate host snail (Pseudosuccinea columella) in uMgungundlovu district, KwaZulu-Natal, South Africa. *BMC Veterinary Research*, *21*(1), 254. <https://doi.org/10.1186/s12917-025-04703-1>
142. Hakyemez, I. N., Aktaş, G., Savli, H., Küçükbayrak, A., Gürel, S., & Taş, T. (2012). A Fascioliasis Case: a not Rare Cause of Hypereosinophilia in Developing Countries, Present in Developed too. *Mediterranean Journal of Hematology and Infectious Diseases*, *4*(1), e2012029. <https://doi.org/10.4084/MJHID.2012.029>
143. Halimi, M., Farajzadeh, M., Delavari, M., & Arbabi, M. (2015). Developing a climate-based risk map of fascioliasis outbreaks in Iran. *Journal of Infection and Public Health*, *8*(5), 481–486. <https://doi.org/10.1016/j.jiph.2015.04.024>
144. Hamel, D., Liu, M., Yoon, S., & Rehbein, S. (2024). Early stage *Fasciola hepatica* infection in growing cattle: impact on weight gain and liver weight determined in flukicide efficacy studies. *Parasitology Research*, *123*(7), 281. <https://doi.org/10.1007/s00436-024-08305-y>
145. Hammami, H., Mezghani-Jarraya, R., Damak, M., & Ayadi, A. (2011). Molluscicidal activity of various solvent extracts from *Solanum nigrum* var. villosum L. aerial parts against *Galba truncatula*. *Parasite*, *18*(1), 63–70. <https://doi.org/10.1051/parasite/2011181063>
146. Hardman, E. W., Jones, R. L., & Davies, A. H. (1970). Fascioliasis--a large outbreak. *British Medical Journal*, *3*(5721), 502–505. https://doi.org/10.1136/bmj.3.5721.502
147. Haridy, F. M., & Morsy, T. A. (2000). Camel: a new Egyptian host for *Fasciola gigantica*. *Journal of the Egyptian Society of Parasitology*, *30*(2), 451–454.
148. Haridy, F. M., Ibrahim, B. B., Morsy, T. A., & El-Sharkawy, I. M. (1999). Fascioliasis an increasing zoonotic disease in Egypt. *Journal of the Egyptian Society of Parasitology*, *29*(1), 35–48.
149. Harinasuta, T., Pungpak, S., & Keystone, J. S. (1993). Trematode infections. Opisthorchiasis, clonorchiasis, fascioliasis, and paragonimiasis. *Infectious Disease Clinics of North America*, *7*(3), 699–716.
150. Haroun, E. M., & Hussein, M. F. (1975). Clinico-pathological studies on naturally-occurring bovine fascioliasis in the Sudan. *Journal of Helminthology*, *49*(3), 143–152.
151. Hecker, A. S., Raulf, M.-K., König, S., Knubben-Schweizer, G., Wenzel, C., May, K., & Strube, C. (2024). In-herd prevalence of *Fasciola hepatica* and Calicophoron / *Paramphistomum* spp. infections in German dairy cows with comparison of two coproscopical methods and establishment of real-time pyrosequencing for rumen fluke species differentiation. *Veterinary Parasitology*, *327*. <https://doi.org/10.1016/j.vetpar.2024.110142>
152. Hillyer, G. V., Sanchez, Z., & de Leon, D. (1985). Immunodiagnosis of bovine fascioliasis by enzyme-linked immunosorbent assay and immunoprecipitation methods. *The Journal of Parasitology*, *71*(4), 449–454.
153. Hörchner, F., Grelck, H., & Flasshoff, F. G. (1976). [Diagnosis of cattle fascioliasis]. *Berliner Und Munchener Tierarztliche Wochenschrift*, *89*(15), 296–300.
154. Hörchner, F., Hennings, R., Verspohl, F., Averbeck, W., & Boch, J. (1970). [Drug control of cattle fascioliasis in the Steinfurt area. II. Results of the 3-year treatment program]. *Berliner Und Munchener Tierarztliche Wochenschrift*, *83*(2), 21–Mereminskiĭ, A. I., & Mazur, P. L. (1973). [Worming of sheep in the acute course of fascioliasis]. *Veterinariia*, *49*(2), 63–64.26.
155. Hosie, B. D. (1985). Use of anthelmintics to control fascioliasis in sheep in the Yemen Arab Republic. *Tropical Animal Health and Production*, *17*(4), 248–251. <https://doi.org/10.1007/BF02356989>
156. Hotez, P. J., Savioli, L., & Fenwick, A. (2012). Neglected tropical diseases of the Middle East and North Africa: review of their prevalence, distribution, and opportunities for control. *PLoS Neglected Tropical Diseases*, *6*(2), e1475. <https://doi.org/10.1371/journal.pntd.0001475>
157. Howell, A. K., & Williams, D. J. L. (2020). The Epidemiology and Control of Liver Flukes in Cattle and Sheep. *Veterinary Clinics of North America: Food Animal Practice*, *36*(1), 109–123. https://doi.org/10.1016/j.cvfa.2019.12.002
158. Howell, A. K., Malalana, F., Beesley, N. J., Hodgkinson, J. E., Rhodes, H., Sekiya, M., Archer, D., Clough, H. E., Gilmore, P., & Williams, D. J. L. (2020). Fasciola hepatica in UK horses. *Equine Veterinary Journal*, *52*(2), 194–199. <https://doi.org/10.1111/evj.13149>
159. Howell, A. K., Malalana, F., Beesley, N. J., Hodgkinson, J. E., Rhodes, H., Sekiya, M., Archer, D., Clough, H. E., Gilmore, P., & Williams, D. J. L. (2020). Fasciola hepatica in UK horses. *Equine Veterinary Journal*, *52*(2), 194–199. <https://doi.org/10.1111/evj.13149>
160. Howell, A., Baylis, M., Smith, R., Pinchbeck, G., & Williams, D. (2015). Epidemiology and impact of Fasciola hepatica exposure in high-yielding dairy herds. *Preventive Veterinary Medicine*, *121*(1-2), 41–48. <https://doi.org/10.1016/j.prevetmed.2015.05.013>
161. Howell, A., Mugisha, L., Davies, J., LaCourse, E. J., Claridge, J., Williams, D. J. L., Kelly-Hope, L., Betson, M., Kabatereine, N. B., & Stothard, J. R. (2012). Bovine fasciolosis at increasing altitudes: parasitological and malacological sampling on the slopes of Mount Elgon, Uganda. *Parasites & Vectors*, *5*, 196. <https://doi.org/10.1186/1756-3305-5-196>
162. Hugho, E. A., Nagagi, Y. P., Lyaruu, L. J., Mosha, V. V., Senyael, N., Mwita, M. M., Mabahi, R. W., Temba, V. M., Hebel, M., Nyati, M., Mmbaga, B. T., Ndyetabura, T. O., & Lukambagire, A. S. (2025). Inverted Patterns of Schistosomiasis and Fascioliasis and Risk Factors Among Humans and Livestock in Northern Tanzania. *Pathogens (Basel, Switzerland)*, *14*(1). <https://doi.org/10.3390/pathogens14010087>
163. Hussein, M. F., & Haroun, E. M. (1977). Pulmonary fascioliasis in Sudanese cattle. *The British Veterinary Journal*, *133*(3), 316–317. <https://doi.org/10.1016/s0007-1935(17)34100-3>
164. Hussein, N. G. A., Mahmoud, M. M., Elsayad, M. H., & Abdel-Tawab, S. M. (2004). Human chronic fascioliasis: a possible cause of unexplained abnormal liver tests. *British Journal of Biomedical Science*, *61*(4), 211–214. <https://doi.org/10.1080/09674845.2004.11732674>
165. Ibarra-Velarde, F., Vera-Montenegro, Y., Nájera-Fuentes, R., & Sánchez-Albarran, A. (2001). Efficacy of combined chemotherapy against gastrointestinal nematodes and *Fasciola hepatica* in cattle. *Veterinary Parasitology*, *99*(3), 199–204. https://doi.org/10.1016/s0304-4017(01)00460-5
166. Ichikawa-Seki, M., Shiroma, T., Kariya, T., Nakao, R., Ohari, Y., Hayashi, K., & Fukumoto, S. (2017). Molecular characterization of *Fasciola* flukes obtained from wild sika deer and domestic cattle in Hokkaido, Japan. *Parasitology International*, *66*(5), 519–521. <https://doi.org/10.1016/j.parint.2017.04.005>
167. Isah, U. M. (2019). Studies on the prevalence of fascioliasis among ruminant animals in northern Bauchi state, north-eastern Nigeria. *Parasite Epidemiology and Control*, *5*. <https://doi.org/10.1016/j.parepi.2019.e00090>
168. Isra’a M. Essa, & Ghazi Y. Azzal. (2024). Occurrence and molecular phylogeny of *Fasciola* species in camels of southwestern Iraq. *Veterinary World*, *17*(12), 2957–2966. <https://doi.org/10.14202/vetworld.2024.2957-2966>
169. Itagaki, T., Ohta, N., Hosaka, Y., Iso, H., Konishi, M., Chinone, S., & Itagaki, H. (1989). Diagnosis of *Fasciola* sp. infections in cattle by enzyme-linked immunosorbent assay. *Nihon Juigaku Zasshi. The Japanese Journal of Veterinary Science*, *51*(4), 757–764. <https://doi.org/10.1292/jvms1939.51.757>
170. Jaja, I. F., Mushonga, B., Green, E., & Muchenje, V. (2017). *Financial loss estimation of bovine fasciolosis in slaughtered cattle in South Africa*. *2*(4), 27–34. <https://doi.org/10.1016/j.parepi.2017.10.001>
171. Johns, D. R., & Dickeson, S. J. (1979). Efficacy of albendazole against Fasciola hepatica in sheep. *Australian Veterinary Journal*, *55*(9), 431–432. <https://doi.org/10.1111/j.1751-0813.1979.tb05599.x>
172. Kabaalioğlu, A., Ceken, K., Saba, R., Artan, R., Cevikol, C., & Yilmaz, S. (2003). Pediatric fascioliasis: report of three cases. *The Turkish Journal of Pediatrics*, *45*(1), 51–54.
173. Kamardinov, K. K. (1985). [Human fascioliasis]. *Meditsinskaia Parazitologiia I Parazitarnye Bolezni*, *5*, 17–20.
174. Kaset, C., Eursitthichai, V., Vichasri-Grams, S., Viyanant, V., & Grams, R. (2010). Rapid identification of lymnaeid snails and their infection with Fasciola gigantica in Thailand. *Experimental Parasitology*, *126*(4), 482–488. <https://doi.org/10.1016/j.exppara.2010.05.021>
175. Kenne, E. L., Yetchom-Fondjo, J. A., Moumite, M. B., Tsekane, S. J., Ngamaleu-Siewe, B., Fouelifack-Nintidem, B., Biawa-Kagmegni, M., Tuekam Kowa, P. S., Fantio, R. M., Yomon, A. K., Kentsop-Tsafong, R. M., Dim-Mbianda, A. M., & Kenne, M. (2021). Species composition and diversity of freshwater snails and land snails at the swampy areas and streams edges in the urban zone of Douala, Cameroon. *International Journal of Biological and Chemical Sciences*, *15*(4), 1297–1324. <https://doi.org/10.4314/ijbcs.v15i4.2>
176. Keyyu, J. D., Kassuku, A. A., Kyvsgaard, N. C., & Monrad, J. (2009). Effectiveness of a community-based strategic anthelmintic treatment programme in the control of gastrointestinal nematodes and *Fasciola gigantica* in cattle in Kilolo district, Tanzania. *Veterinary Research Communications : An International Journal Publishing Topical Reviews and Research Articles on All Aspects of the Veterinary Sciences*, *33*(6), 555–565. https://doi.org/10.1007/s11259-009-9204-9
177. Keyyu, J. D., Kyvsgaard, N. C., Monrad, J., & Kassuku, A. A. (2009). Effectiveness of strategic anthelmintic treatments in the control of gastrointestinal nematodes and *Fasciola gigantica* in cattle in Iringa region, Tanzania. *Tropical Animal Health and Production*, *41*(1), 25–33. <https://doi.org/10.1007/s11250-008-9150-5>
178. Khademvatan, S., Majidiani, H., Khalkhali, H., Taghipour, A., Asadi, N., & Yousefi, E. (2019). Prevalence of fasciolosis in livestock and humans: A systematic review and meta-analysis in Iran. *Comparative Immunology, Microbiology and Infectious Diseases*, *65*, 116–123. <https://doi.org/10.1016/j.cimid.2019.05.001>
179. Khan, M. K., Sajid, M. S., Riaz, H., Ahmad, N. E., He, L., Shahzad, M., Hussain, A., Khan, M. N., Iqbal, Z., & Zhao, J. (2013). The global burden of fasciolosis in domestic animals with an outlook on the contribution of new approaches for diagnosis and control. *Parasitology Research*, *112*(7), 2421–2430. <https://doi.org/10.1007/s00436-013-3464-6>
180. Khan, M., Anisur Rahman, A. K. M., Ahsan, S., Ehsan, A., Dhand, N., & Ward, M. P. (2017). Bovine fascioliasis risk factors and space-time clusters in Mymensingh, Bangladesh. *Veterinary Parasitology: Regional Studies and Reports*, *9*, 104–109. <https://doi.org/10.1016/j.vprsr.2017.06.007>
181. Khedr, A. A., Abdel-Hakeem, S. S., Mahmoud, W. G., Abdel-Hakeem, S. S., Al-Hakami, A. M., Tolba, M. E. M., Abd-Elrahman, S. M., & Khalifa, M. M. (2025). An overview of fasciolosis in human and cattle populations in New Valley, Egypt. *Frontiers in Veterinary Science*, *12*. <https://doi.org/10.3389/fvets.2025.1572946>
182. King, T., Cabada, M. M., Kowalewska-Grochowska, K., & Vaughan, S. D. (2024). Eosinophilic Liver Abscess: A Case of Locally Acquired *Fasciola hepatica* in Alberta, Canada. *Open Forum Infectious Diseases*, *11*(3). <https://doi.org/10.1093/ofid/ofae101>
183. Knottenbelt, D. C. (1974). Some pathological findings in wild bushbuck (Tragelaphus scriptus). *The Veterinary Record*, *94*(9), 173. <https://doi.org/10.1136/vr.94.9.173>
184. Kobuleĭ, T. (1969). [Controlling fascioliasis]. *Veterinariia*, *46*(7), 105–106.
185. Kordshooli, M. S., Solhjoo, K., Armand, B., Dowlatkhah, H., & Jahromi, M. E. (2017). A reducing trend of fasciolosis in slaughtered animals based on abattoir data in South of Iran. *Veterinary World*, *10*(4), 418–423. <https://doi.org/10.14202/vetworld.2017.418-423>
186. Kosaka, S., Shimizu, S., Nakayamada, S., Nawata, A., Shimono, N., Tanaka, M., Maruyama, H., Osada, Y., & Tanaka, Y. (2024). A delayed diagnosis of fascioliasis: The importance of appropriate fecal diagnostic method. *Journal of Infection and Chemotherapy : Official Journal of the Japan Society of Chemotherapy*, *30*(5), 454–458. <https://doi.org/10.1016/j.jiac.2023.11.006>
187. Köstenberger, K., Tichy, A., Bauer, K., Pless, P., & Wittek, T. (2017). Associations between fasciolosis and milk production, and the impact of anthelmintic treatment in dairy herds. *Parasitology Research : Founded as Zeitschrift Für Parasitenkunde*, *116*(7), 1981–1987. <https://doi.org/10.1007/s00436-017-5481-3>
188. Kronenberg, P. A., Fouché, N., Sekiya, M., Liechti, P., Frey, C. F., Mulcahy, G., & Eichenberger, R. M. (2025). A serological test based on mutated recombinant *Fasciola hepatica* cathepsin L protease for the diagnosis of equine fasciolosis. *Veterinary Parasitology*, *340*. <https://doi.org/10.1016/j.vetpar.2025.110592>
189. Kumar, N., Ghosh, S., & Gupta, S. C. (2008). Detection of *Fasciola gigantica* infection in buffaloes by enzyme-linked immunosorbent assay. *Parasitology Research*, *104*(1), 155–161. <https://doi.org/10.1007/s00436-008-1174-2>
190. Kumar, V. (1989). Bovine fascioliasis: a need to revisit control strategy. *The Veterinary Record*, *124*(7), 178.
191. Laird, P. P., & Boray, J. C. (1992). Human fascioliasis successfully treated with triclabendazole. *Australian and New Zealand Journal of Medicine*, *22*(1), 45–47. <https://doi.org/10.1111/j.1445-5994.1992.tb01708.x>
192. Lalrinkima, H., Lalchhandama, C., Jacob, S. S., Raina, O. K., & Lallianchhunga, M. C. (2021). Fasciolosis in India: An overview. *Experimental Parasitology*, *222*. https://doi.org/10.1016/j.exppara.2021.108066
193. Lalrinkima, H., Lalchhandama, C., Jacob, S. S., Raina, O. K., & Lallianchhunga, M. C. (2021). Fasciolosis in India: An overview. *Experimental Parasitology*, *222*. <https://doi.org/10.1016/j.exppara.2021.108066>
194. LaPook, J. D., Magun, A. M., Nickerson, K. G., & Meltzer, J. I. (2000). Sheep, watercress, and the Internet. *Lancet (London, England)*, *356*(9225), 218. <https://doi.org/10.1016/S0140-6736(00)02484-3>
195. Leathers, C. W., Foreyt, W. J., Fetcher, A., & Foreyt, K. M. (1982). Clinical fascioliasis in domestic goats in Montana. *Journal of the American Veterinary Medical Association*, *180*(12), 1451–1454.
196. Lebacq, E., Gilon, L., Desmet, V., & Van Meirvenne, N. (1971). [Liver changes induced by *Fasciola hepatica* in humans]. *Acta Gastro-Enterologica Belgica*, *34*(3), 322–328.
197. Leerapun, A., Puasripun, S., Kijdamrongthum, P., & Thongsawat, S. (2021). Human fascioliasis presenting as liver abscess: clinical characteristics and management. *Hepatology International*, *15*(3), 804–811. <https://doi.org/10.1007/s12072-021-10180-z>
198. Lemma, B., Gabre-ab, F., & Tedla, S. (1985). Studies on fascioliasis in four selected sites in Ethiopia. *Veterinary Parasitology*, *18*(1), 29–37. <https://doi.org/10.1016/0304-4017(85)90005-6>
199. Levy, M. G., Tunis, M., & Isseroff, H. (1973). Population control in snails by natural inhibitors. *Nature*, *241*(5384), 65–66. <https://doi.org/10.1038/241065a0>
200. Lu, X.-T., Gu, Q.-Y., Limpanont, Y., Song, L.-G., Wu, Z.-D., Okanurak, K., & Lv, Z.-Y. (2018). Snail-borne parasitic diseases: an update on global epidemiological distribution, transmission interruption and control methods. *Infectious Diseases of Poverty*, *7*. <https://doi.org/10.1186/s40249-018-0414-7>
201. Luis, H. C., Matias, F. B. R., Tubalinal, G. A. S., & Mingala, C. N. (2021). In vitro anthelminthic activity of fringed spiderflower (Cleome rutidosperma) ethanolic leaf extract against *Fasciola* spp. *Annals of Parasitology*, *67*(2), 243–248. <https://doi.org/10.17420/ap6702.335>
202. Lukambagire, A.-H. S., Mchaile, D. N., & Nyindo, M. (2015). Diagnosis of human fascioliasis in Arusha region, northern Tanzania by microscopy and clinical manifestations in patients. *BMC Infectious Diseases*, *15*, 578. <https://doi.org/10.1186/s12879-015-1326-9>
203. Luz, J., Focaccia Siciliano R, de Oliveira, A., & Pisani, J. (1999). Human Fascioliasis in The Metropolitan Area of Curitiba, Brazil Evaluation of The Foci of Infection and Report of Nine Cases Treated With Triclabendazole. *The Brazilian Journal of Infectious Diseases : An Official Publication of the Brazilian Society of Infectious Diseases*, *3*(6), 220–225.
204. Lyngdoh, D., Sharma, S., Roy, B., & Tandon, V. (2016). Animal Fascioliasis: Perspectives from high altitudinal regions. *Veterinary Parasitology*, *232*, 21–31. <https://doi.org/10.1016/j.vetpar.2016.11.007>
205. Machicado, C., Machicado, J. D., Maco, V., Terashima, A., Marcos, L. A., & Garcia, H. H. (2016). Association of Fasciola hepatica Infection with Liver Fibrosis, Cirrhosis, and Cancer: A Systematic Review. *PLOS Neglected Tropical Diseases*, *10*(9). <https://doi.org/10.1371/journal.pntd.0004962>
206. Magalhães, K. G., Passos, L. K. J., & Carvalho, O. d. S. (2004). Detection of Lymnaea columella infection by *Fasciola hepatica* through Multiplex-PCR. *Memorias Do Instituto Oswaldo Cruz*, *99*(4), 421–424. <https://doi.org/10.1590/s0074-02762004000400013>
207. Mahato, S. N., Harrison, L. J., & Hammond, J. A. (1994). Efficacy of triclabendazole against *Fasciola gigantica* in buffaloes in eastern Nepal. *The Veterinary Record*, *135*(24), 579–580.
208. Mahato, S., & Harrison, L. (2005). Control of Fasciolosis in Stall-fed Buffaloes by Managing the Feeding of Rice Straw. *Tropical Animal Health and Production*, *37*(4), 285–291.
209. Mahdi, N. K., & Al-Baldawi, F. A. (1987). Hepatic fascioliasis in the abattoirs of Basrah. *Annals of Tropical Medicine and Parasitology*, *81*(4), 377–379. <https://doi.org/10.1080/00034983.1987.11812135>
210. Malatji, M. P., Pfukenyi, D. M., & Mukaratirwa, S. (2019). *Fasciola* species and their vertebrate and snail intermediate hosts in East and Southern Africa: a review. *Journal of Helminthology*, *94*, e63. <https://doi.org/10.1017/S0022149X19000531>
211. Malone, J. B. (1986). Fascioliasis and cestodiasis in cattle. *The Veterinary Clinics of North America. Food Animal Practice*, *2*(2), 261–275.
212. Marcos, L., Maco, V., & Terashima, A. (2021). Triclabendazole for the treatment of human fascioliasis and the threat of treatment failures. *Expert Review of Anti-Infective Therapy*, *19*(7), 817–823. <https://doi.org/10.1080/14787210.2021.1858798>
213. Marques, S. M. T., Scroferneker, M. L., & Edelweiss, M. I. A. (2004). Glomerulonephritis in water buffaloes (*Bubalus bubalis*) naturally infected by Fasciola hepatica. *Veterinary Parasitology*, *123*(1-2), 83–91. <https://doi.org/10.1016/j.vetpar.2004.06.011>
214. Martínez-Sernández, V., Muiño, L., Perteguer, M. J., Gárate, T., Mezo, M., González-Warleta, M., Muro, A., Correia da Costa, J. M., Romarís, F., & Ubeira, F. M. (2011). Development and evaluation of a new lateral flow immunoassay for serodiagnosis of human fasciolosis. *PLoS Neglected Tropical Diseases*, *5*(11), e1376. <https://doi.org/10.1371/journal.pntd.0001376>
215. Martínez-Valladares, M., & Rojo-Vázquez, F. A. (2016). Loop-mediated isothermal amplification (LAMP) assay for the diagnosis of fasciolosis in sheep and its application under field conditions. *Parasites & Vectors*, *9*, 73. <https://doi.org/10.1186/s13071-016-1355-2>
216. Martínez-Valladares, M., Cordero-Pérez, C., Castañón-Ordóñez, L., Famularo, M. R., Fernández-Pato, N., & Rojo-Vázquez, F. A. (2010). Efficacy of a moxidectin/triclabendazole oral formulation against mixed infections of Fasciola hepatica and gastrointestinal nematodes in sheep. *Veterinary Parasitology*, *174*(1-2), 166–169. <https://doi.org/10.1016/j.vetpar.2010.07.008>
217. Mas-Coma, S. (2005). Epidemiology of fascioliasis in human endemic areas. *Journal of Helminthology*, *79*(3), 207–216. <https://doi.org/10.1079/joh2005296>
218. Mas-Coma, S., Bargues, M. D., & Valero, M. A. (2005). Fascioliasis and other plant-borne trematode zoonoses. *International Journal for Parasitology*, *35*(11-12), 1255–1278. <https://doi.org/10.1016/j.ijpara.2005.07.010>
219. Mas-Coma, S., Bargues, M. D., & Valero, M. A. (2014). Diagnosis of human fascioliasis by stool and blood techniques: update for the present global scenario. *Parasitology*, *141*(14), 1918–1946. <https://doi.org/10.1017/S0031182014000869>
220. Mas-Coma, S., Valero, M. A., & Bargues, M. D. (2009). Chapter 2. Fasciola, lymnaeids and human fascioliasis, with a global overview on disease transmission, epidemiology, evolutionary genetics, molecular epidemiology and control. *Advances in Parasitology*, *69*, 41–146. https://doi.org/10.1016/S0065-308X(09)69002-3
221. Mas-Coma, S., Valero, M. A., & Bargues, M. D. (2019). Fascioliasis. In [Editor(s) if applicable, or omit if none provided], Advances in Experimental Medicine and Biology (Vol. 1154, pp. 71–103).
222. Mas-Coma, S., Valero, M. A., & Bargues, M. D. (2022). Human and Animal Fascioliasis: Origins and Worldwide Evolving Scenario. *Clinical Microbiology Reviews*, *35*(4). https://doi.org/10.1128/cmr.00088-19
223. Mas-Coma, S., Valero, M. A., & Bargues, M. D. (2023). One Health for fascioliasis control in human endemic areas. *Trends in Parasitology*, *39*(8), 650–667. <https://doi.org/10.1016/j.pt.2023.05.009>
224. Materu, G. S., Nzalawahe, J., Sengupta, M. E., Stensgaard, A.-S., Katakweba, A., Silvester, Y., Mwing'a, G. P., Vennervald, B. J., & Kinung'hi, S. (2025). Epidemiological Survey of Human Zoonotic Fascioliasis and Schistosomiasis in the Lake Victoria and Southern Highland Ecological Zones of Tanzania. *Zoonotic Diseases*, *5*(4), 29. <https://doi.org/10.3390/zoonoticdis5040029>
225. Matin, S., Joukar, F., Yeganeh, S., Daryakar, A., & Mansour-Ghanaei, F. (2022). Liver Abscess due to Fasciola hepatica: A Case Report of the North of Iran. *Case Reports in Infectious Diseases*, *2022*, 4399061. <https://doi.org/10.1155/2022/4399061>
226. Mazeri, S., Rydevik, G., Handel, I., Bronsvoort, B. M. d., & Sargison, N. (2017). Estimation of the impact of Fasciola hepatica infection on time taken for UK beef cattle to reach slaughter weight. *Scientific Reports*, *7*(1), 7319. <https://doi.org/10.1038/s41598-017-07396-1>
227. Meharenet B, & Shitu D. (2021). Concurrent Infection of Fascioliasis andTrypanosomosis and Associated Risk Factors in Local Zebu Breed Cattle of Western Ethiopia. *Veterinary Medicine: Research and Reports*, *12*, 15–22.
228. Mehmood, K., Zhang, H., Sabir, A. J., Abbas, R. Z., Ijaz, M., Durrani, A. Z., Saleem, M. H., Ur Rehman, M., Iqbal, M. K., Wang, Y., Ahmad, H. I., Abbas, T., Hussain, R., Ghori, M. T., Ali, S., Khan, A. U., & Li, J. (2017). A review on epidemiology, global prevalence and economical losses of fasciolosis in ruminants. *Microbial Pathogenesis*, *109*, 253–262. <https://doi.org/10.1016/j.micpath.2017.06.006>
229. Mekky, M. A., Tolba, M., Abdel-Malek, M. O., Abbas, W. A., & Zidan, M. (2015). Human fascioliasis: a re-emerging disease in Upper Egypt. *The American Journal of Tropical Medicine and Hygiene*, *93*(1), 76–79. <https://doi.org/10.4269/ajtmh.15-0030>
230. Mekroud, A., Benakhla, A., Vignoles, P., Rondelaud, D., & Dreyfuss, G. (2004). Preliminary studies on the prevalences of natural fasciolosis in cattle, sheep, and the host snail (Galba truncatula) in north-eastern Algeria. *Parasitology Research*, *92*(6), 502–505. <https://doi.org/10.1007/s00436-004-1072-1>
231. Mendes, T. M. F., Filho, D. V. B., Bataglioli, A. S., do Prado, A. P., Madi, R. R., Ueta, M. T., Pinto, M. C., & Allegretti, S. M. (2019). Bovine fasciolisis in São Paulo state, Brazil. *Veterinary Parasitology, Regional Studies and Reports*, *17*, 100293. <https://doi.org/10.1016/j.vprsr.2019.100293>
232. Mereminskiĭ, A. I. (1967). [The prognosis of fascioliasis and paramphistomiasis in ruminants]. *Veterinariia*, *44*(5), 76–78.
233. Mezo, M., González-Warleta, M., Castro-Hermida, J. A., Manga-González, M. Y., Peixoto, R., Mas-Coma, S., & Valero, M. A. (2013). The wild boar (Sus scrofa Linnaeus, 1758) as secondary reservoir of *Fasciola hepatica* in Galicia (NW Spain). *Veterinary Parasitology*, *198*(3-4), 274–283. <https://doi.org/10.1016/j.vetpar.2013.09.009>
234. Mirzadeh, A., Jafarihaghighi, F., Kazemirad, E., Sabzevar, S. S., Tanipour, M. H., & Ardjmand, M. (2021). Recent Developments in Recombinant Proteins for Diagnosis of Human Fascioliasis. *Acta Parasitologica*, *66*(1), 13–25. <https://doi.org/10.1007/s11686-020-00280-5>
235. Mochankana, M. E., & Robertson, I. D. (2016). A retrospective study of the prevalence of bovine fasciolosis at major abattoirs in Botswana. *The Onderstepoort Journal of Veterinary Research*, *83*(1), a1015. <https://doi.org/10.4102/ojvr.v83i1.1015>
236. Mohamed, D. K. A. (2021). A study on causes of cattle liver condemnation at an abattoir in Omdurman area, Khartoum State, Sudan. *BMC Veterinary Research*, *17*(1). <https://doi.org/10.1186/s12917-021-02766-4>
237. Mohammed, A. (2026). Fascioliasis: A Neglected Zoonotic Disease of Global Public Health Importance. *Alexandria Journal of Veterinary Sciences*, *89*(0), 42. <https://doi.org/10.5455/ajvs.310112>
238. Mohammed, E. S., & Maky, M. A. (2020). Meat Condemnations and Economic Importance in the Northern and Southern Egyptian Abattoirs. *Advances in Animal and Veterinary Sciences*, *8*(1). <https://doi.org/10.17582/journal.aavs/2020/8.1.96.107>
239. Molento, M. B., Bennema, S., Bertot, J., Pritsch, I. C., & Arenal, A. (2018). *Bovine fascioliasis in Brazil: Economic impact and forecasting*. *12*, 1–3. <https://doi.org/10.1016/j.vprsr.2017.12.004>
240. Molina, E., Gonzaga, E., Sinolinding, E., Lumbao, L., Peralta, A., & Barraca, A. (2005). Differences in Susceptibility between Cattle and Swamp Buffaloes to Infection with Fasciola gigantica. *Tropical Animal Health and Production*, *37*(8), 611–616.
241. Molla, D., Nazir, S., Mohammed, A., & Tintagu, T. (2019). Parasitic infections as major cause of abattoir condemnations in cattle slaughtered at an Ethiopian abattoir: 10-year retrospective study. *Journal of Helminthology*, *94*, e31. https://doi.org/10.1017/S0022149X1900004X
242. Mollinedo, S., Gutierrez, P., Azurduy, R., Valle, F., Salas, A., Mollinedo, Z., Soto, P., Villarroel, C. F., Ransom, J., Lawrence, R., Berman, J., & Soto, J. (2019). Mass Drug Administration of Triclabendazole for Fasciola Hepatica in Bolivia. *The American Journal of Tropical Medicine and Hygiene*, *100*(6), 1494–1497. <https://doi.org/10.4269/ajtmh.19-0060>
243. Mora, P., Gea, F., Agud, J. L., Rábago, L., Vargas, E., & De Juana, P. (1992). [Hepatic fascioliasis. Resistance to praziquantel treatment and the radiological findings]. *Revista Espanola de Enfermedades Digestivas*, *82*(3), 197–199.
244. Moshfe, A., Aria, A., Erfani, N., Jamshidi, A., Sarkari, B., Abdolahi Khabisi, S., & Arefkhah, N. (2020). Clinical Features, Diagnosis and Management of Patients with Suspicion of Fascioliasis in Kohgiluyeh and Boyer-Ahmad Province, Southwestern Iran. *Iranian Journal of Parasitology*, *15*(1), 84–90.
245. Mostafa, B. B. (2006). Effect of three dormant oils on schistosomiasis and fascioliasis vector snails and its relation with some non-target snails. *Journal of the Egyptian Society of Parasitology*, *36*(3), 809–826.
246. Mubanga, C., Mwape, K. E., Phiri, I. K., Trevisan, C., Zulu, G., Chabala, C., van Damme, I., Schmidt, V., Dorny, P., & Gabriël, S. (2019). Progress on the development of rapid diagnostic tests for foodborne neglected zoonotic helminthiases: A systematic review. *Acta Tropica*, *194*, 135–147. <https://doi.org/10.1016/j.actatropica.2019.03.030>
247. Mzembe, S. A., & Chaudhry, M. A. (1979). The epidemiology of fascioliasis in Malawi: I. The epidemiology in the intermediate host. *Tropical Animal Health and Production*, *11*(4), 246–250.
248. Mzembe, S. A., & Chaudhry, M. A. (1981). The epidemiology of fascioliasis in Malawi. Part II. Epidemiology in the definitive host. *Tropical Animal Health and Production*, *13*(1), 27–33. <https://doi.org/10.1007/BF02237882>
249. Najib, M. A., Izani, N. J. N., Amilah, W. A. W. W. N., Faez, A. M., & Shafizol, Z. (2020). A Scoping Review of the Prevalence of Fascioliasis in Malaysia and Risk Factors for Infection. *The Malaysian Journal of Medical Sciences : MJMS*, *27*(1), 22–36. <https://doi.org/10.21315/mjms2020.27.1.3>
250. Nambuya, S., Kalinda, C., Vudriko, P., Adriko, M., Phiri, M., Mindu, T., Wagaba, D., & Mugisha, L. (2025). Meta-analysis and systematic review of the prevalence and risk factors of animal fascioliasis in Eastern and Southern Africa between 2000 and 2023. *Preventive Veterinary Medicine*, *239*. <https://doi.org/10.1016/j.prevetmed.2025.106490>
251. Namirembe, D., Huyse, T., Wangalwa, R., Tumusiime, J., & Tolo, C. U. (2024). Liver fluke and schistosome cross-infection risk between livestock and wild mammals in Western Uganda, a One Health approach. *International Journal for Parasitology: Parasites and Wildlife*, *25*. <https://doi.org/10.1016/j.ijppaw.2024.101022>
252. Neira, G., Mera y Sierra, R., Godoy, D., Logarzo, L., Gonzalez, M., & Scarcella, S. (2025). Influence of water quality parameters on lymnaeid snail distribution and their relationship with fasciolosis transmission. *Veterinary Parasitology*, *340*. <https://doi.org/10.1016/j.vetpar.2025.110611>
253. Nguyen Thu, H., Dermauw, V., Tran Huy, T., Roucher, C., Dorny, P., Nguyen Thi, H., Trung, K. H., Dao Van, T., Do Nhu, B., & Nguyen Kim, T. (2022). Diagnosing Human Fascioliasis Using ELISA Immunoassays at a Tertiary Referral Hospital in Hanoi: A Cross-Sectional Study. *Tropical Medicine and Infectious Disease*, *7*(5). <https://doi.org/10.3390/tropicalmed7050076>
254. Nguyen, T., Cheong, F. W., Liew, J. W. K., & Lau, Y. L. (2016). Seroprevalence of fascioliasis, toxocariasis, strongyloidiasis and cysticercosis in blood samples diagnosed in Medic Medical Center Laboratory, Ho Chi Minh City, Vietnam in 2012. *Parasites & Vectors*, *9*(1). <https://doi.org/10.1186/s13071-016-1780-2>
255. Njau, B. C., & Scholtens, R. G. (1991). The role of traditionally harvested hay in the transmission of ovine fasciolosis in the Ethiopian highlands. *Veterinary Research Communications*, *15*(5), 369–372.
256. Njau, B. C., Kasali, O. B., Scholtens, R. G., & Akalework, N. (1989). The influence of watering practices on the transmission of *Fasciola* among sheep in the Ethiopian highlands. *Veterinary Research Communications*, *13*(1), 67–74. <https://doi.org/10.1007/BF00366854>
257. Njeh, F., Feki, H., Koubaa, I., Hamed, N., Damak, M., Ayadi, A., Hammami, H., & Mezghani-Jarraya, R. (2016). Molluscicidal activity of *Solanum elaeagnifolium* seeds against *Galba truncatula* intermediate host of *Fasciola hepatica:* Identification of β-solamarine. *Pharmaceutical Biology*, *54*(4), 726–731. <https://doi.org/10.3109/13880209.2015.1073332>
258. Nkurunziza, S., Nishemezwe, G., Ntirandekura, J.-B., Niyokwizera, P., Nyabongo, L., Omore, A., & Odhiambo, R. (2023). Prevalence and associated risk factors assessment of bovine fasciolosis in the Imbo Region, Burundi. *Parasitology Research*, *123*(1), 33. <https://doi.org/10.1007/s00436-023-08040-w>
259. Novobilský, A., Sollenberg, S., & Höglund, J. (2015). Distribution of *Fasciola hepatica* in Swedish dairy cattle and associations with pasture management factors. *Geospatial Health*, *9*(2), 293–300. <https://doi.org/10.4081/gh.2015.351>
260. Ntivuguruzwa, J. B., Ntampaka, P., Twagirayezu, E., Isingizwe, S., Habumugisha, J., Mbonyinshuti, V., Bariyanga, J. D., Tumusiime, M., Manirarora, V., Sibomana, E., Mbonigaba, J. B., Hugho, E. A., Lukambagire, A. S., & Ntawubizi, M. (2026). One Health assessment of fascioliasis and schistosomiasis in humans, livestock, and snail vectors in peri-urban Rwanda. *Veterinary World*, *19*(3), 1368–1386. <https://doi.org/10.14202/vetworld.2026.1368-1386>
261. Nur Hafizah, S., Noor Izani, N. J., Ahmad Najib, M., & Wan-Nor-Amilah, W. A. W. (2023). Immunodiagnosis of Fascioliasis in Ruminants by ELISA Method: A Mini-Review. *The Malaysian Journal of Medical Sciences : MJMS*, *30*(4), 25–32. <https://doi.org/10.21315/mjms2023.30.4.3>
262. Nyindo, M., & Lukambagire, A.-H. (2015). Fascioliasis: An Ongoing Zoonotic Trematode Infection. *BioMed Research International*, *2015*, 786195. <https://doi.org/10.1155/2015/786195>
263. Nyirenda, S. S., Sakala, M., Moonde, L., Kayesa, E., Fandamu, P., Banda, F., & Sinkala, Y. (2019). Prevalence of bovine fascioliasis and economic impact associated with liver condemnation in abattoirs in Mongu district of Zambia. *BMC Veterinary Research*, *15*(1), 33. https://doi.org/10.1186/s12917-019-1777-0
264. Nyirenda, S. S., Sakala, M., Moonde, L., Kayesa, E., Fandamu, P., Banda, F., & Sinkala, Y. (2019). Prevalence of bovine fascioliasis and economic impact associated with liver condemnation in abattoirs in Mongu district of Zambia. *BMC Veterinary Research*, *15*(1), 1–8. <https://doi.org/10.1186/s12917-019-1777-0>
265. Nzalawahe, J., Kassuku, A. A., Stothard, J. R., Coles, G. C., & Eisler, M. C. (2014). Trematode infections in cattle in Arumeru District, Tanzania are associated with irrigation. *Parasites & Vectors*, *7*, 107. https://doi.org/10.1186/1756-3305-7-107
266. Odeniran, P. O., & Ademola, I. O. (2016). Zoonotic Parasites of Wildlife in Africa: A Review. *South African Journal of Wildlife Research*, *46*(1), 1–13. <https://doi.org/10.3957/056.046.0001>
267. Odeniran, P. O., Omolabi, K. F., & Ademola, I. O. (2021). Economic impact assessment of small ruminant fasciolosis in Nigeria using pooled prevalence obtained from literature and field epidemiological data. *Veterinary Parasitology: Regional Studies and Reports*, *24*. <https://doi.org/10.1016/j.vprsr.2021.100548>
268. Ogunrinade, A. F., Adenaike, F. A., Fajinmi, J. L., & Bamgboye, E. A. (1980). Bovine fascioliasis in Nigeria. II. Blood chemistry and its correlation with worm burdens in chronicc fascioliasis. *Zentralblatt Fur Veterinarmedizin. Reihe B. Journal of Veterinary Medicine. Series B*, *27*(8), 622–630.
269. Ogurinade, A., & Ogunrinade, B. I. (1980). Economic importance of bovine fascioliasis in Nigeria. *Tropical Animal Health and Production*, *12*(3), 155–160. <https://doi.org/10.1007/BF02242647>
270. Opio, L. G., Abdelfattah, E. M., Terry, J., Odongo, S., Okello, E., Manfredi, M. T., Gazzonis, A. L., Mortarino, M., Villa, L., & Zanzani, S. (2021). Prevalence of Fascioliasis and Associated Economic Losses in Cattle Slaughtered at Lira Municipality Abattoir in Northern Uganda. *Animals : An Open Access Journal from MDPI*, *11*(3). <https://doi.org/10.3390/ani11030681>
271. Osman, M. M., Shehab, A. Y., Zaki, A., & Farag, H. F. (2011). Evaluation of two doses of triclabendazole in treatment of patients with combined schistosomiasis and fascioliasis. *Eastern Mediterranean Health Journal = La Revue de Sante de La Mediterranee Orientale = Al-Majallah Al-Sihhiyah Li-Sharq Al-Mutawassit*, *17*(4), 266–270.
272. Ouchene-Khelifi, N. A., Ouchene, N., Dahmani, H., Dahmani, A., Sadi, M., & Douifi, M. (2018). Fasciolosis due to *Fasciola hepatica* in ruminants in abattoirs and its economic impact in two regions in Algeria. *Tropical Biomedicine*, *35*(1), 181–187.
273. Oyeyemi, O. T., Fasomoyin, E. A., Oriade, T. O., Sulaiman, K. A., Babatunde, O. S., & Oyeyemi, I. T. (2025). Molluscicidal activities of Senna alata silver nanoparticles against adult and egg stages of *Lymnaea natalensis*. *Scientific Reports*, *15*(1), 25746. <https://doi.org/10.1038/s41598-025-11771-8>
274. Pan, M., Bai, S.-Y., Ji, T.-K., Fan, Y.-M., Liu, D.-D., Yang, Y., Tao, J.-P., & Huang, S.-Y. (2022). Epidemiology of *Fasciola* spp. in the intermediate host in China: A potential risk for fasciolosis transmission. *Acta Tropica*, *230*. https://doi.org/10.1016/j.actatropica.2022.106394
275. Pereira, S., Cerqueira, P., Azevedo, S., Sousa, B., & Faraldo, S. (2024). Human Fascioliasis in Portugal: A Case Report. *Cureus*, *16*(11), e74527. https://doi.org/10.7759/cureus.74527
276. Periago, M. V., Valero, M. A., Artigas, P., Agramunt, V. H., Bargues, M. D., Curtale, F., & Mas-Coma, S. (2021). Very High Fascioliasis Intensities in Schoolchildren from Nile Delta Governorates, Egypt: The Old World Highest Burdens Found in Lowlands. *Pathogens (Basel, Switzerland)*, *10*(9). <https://doi.org/10.3390/pathogens10091210>
277. Pfister, K. (1990). Serodiagnosis of fasciolosis in ruminants. *Revue Scientifique et Technique (International Office of Epizootics)*, *9*(2), 511–518. <https://doi.org/10.20506/rst.9.2.499>
278. Pfukenyi, D. M., Monrad, J., & Mukaratirwa, S. (2005). Epidemiology and control of trematode infections in cattle in Zimbabwe: a review. *Journal of the South African Veterinary Association*, *76*(1), 9–17. <https://doi.org/10.4102/jsava.v76i1.387>
279. Phiri, A. M., Phiri, I. K., Sikasunge, C. S., & Monrad, J. (2005). Prevalence of fasciolosis in Zambian cattle observed at selected abattoirs with emphasis on age, sex and origin. *Journal of Veterinary Medicine. B, Infectious Diseases and Veterinary Public Health*, *52*(9), 414–416. <https://doi.org/10.1111/j.1439-0450.2005.00872.x>
280. Pratiwi, K. W., Koesdarto, S., & Harijani, N. (2019). Fasciolosis Prevalence on Several cattle Breeds. *Indian Journal of Public Health Research & Development*, *10*(9), 1478. <https://doi.org/10.5958/0976-5506.2019.02657.3>
281. Priddle, J., Mengersen, K., Swindells, D., Elliott, T., & Ralph, C. (2025). Carcass weight, meat quality and economic impact of liver fluke infection on cattle. *Veterinary Parasitology: Regional Studies and Reports*, *58*. <https://doi.org/10.1016/j.vprsr.2025.101195>
282. Pulpeiro, J. R., Armesto, V., Varela, J., & Corredoira, J. (1991). Fascioliasis: findings in 15 patients. *The British Journal of Radiology*, *64*(765), 798–801. https://doi.org/10.1259/0007-1285-64-765-798
283. Rajasekariah, G. R., & Howell, M. J. (1981). Age-associated responses in susceptible and resistant rats to infection with *Fasciola hepatica*. *International Journal for Parasitology*, *11*(1), 59–65. <https://doi.org/10.1016/0020-7519(81)90026-6>
284. Ramisz, A., & Urban, E. (1968). The effectiveness of the sedimentation method in diagnosis of Fasciola hepatica in cattle. *Wiadomosci Parazytologiczne*, *14*(5), 713–714.
285. Reid, J. F., Doyle, J. J., Armour, J., & Jennings, F. W. (1972). Fasciola hepatica infection in cattle. *The Veterinary Record*, *90*(17), 486–487. https://doi.org/10.1136/vr.90.17.486
286. Riazi, H. (2001). Large liver abscess: unusual presentation of fascioliasis: case report and literature review. *Saudi Journal of Gastroenterology: Official Journal of the Saudi Gastroenterology Association*, *7*(3), 113–115.
287. Righi, S., Benakhla, A., Mekroud, A., Ouchene, N., & Sedraoui, S. (2016). Prevalence of *Fasciola hepatica* in *Galba truncatula* detected by Multiplex PCR in the province of El Tarf (Algeria). *Tropical Biomedicine*, *33*(1), 149–158.
288. Rinaldi, L., Gonzalez, S., Guerrero, J., Aguilera, L. C., Musella, V., Genchi, C., & Cringoli, G. (2012). A One-Health integrated approach to control fascioliasis in the Cajamarca valley of Peru. *Geospatial Health*, *6*(3), S67–73. <https://doi.org/10.4081/gh.2012.124>
289. Roberts, J. A., & Suhardono. (1996). Approaches to the control of fasciolosis in ruminants. *International Journal for Parasitology*, *26*(8-9), 971–981. <https://doi.org/10.1016/s0020-7519(96)80074-9>
290. Rodriguez Quinteros, A. C., Soler, P., Larroza, M., Morales, J. M., & Gurevitz, J. M. (2024). Water temperature and microenvironmental factors predict the presence and detection of the snail host of *Fasciola hepatica* in Andean Patagonia. *Veterinary Parasitology*, *329*. <https://doi.org/10.1016/j.vetpar.2024.110209>
291. Roessler, A. S., Oehm, A. W., Knubben-Schweizer, G., & Groll, A. (2022). A machine learning approach for modelling the occurrence of *Galba truncatula* as the major intermediate host for *Fasciola hepatica* in Switzerland. *Preventive Veterinary Medicine*, *200*. <https://doi.org/10.1016/j.prevetmed.2022.105569>
292. Rondelaud, D., & Vareille-Morel, C. (1994). The chemical and biological control of *Lymnaea truncatula* in natural watercress beds in the Limousin region (France). *Parasite (Paris, France)*, *1*(1), 89–92. <https://doi.org/10.1051/parasite/19940110897>
293. Rondelaud, D., Hourdin, P., Vignoles, P., & Dreyfuss, G. (2005). The contamination of wild watercress with *Fasciola hepatica* in central France depends on the ability of several lymnaeid snails to migrate upstream towards the beds. *Parasitology Research*, *95*(5), 305–309. <https://doi.org/10.1007/s00436-004-1283-5>
294. Rondelaud, D., Vignoles, P., Abrous, M., & Dreyfuss, G. (2001). The definitive and intermediate hosts of *Fasciola hepatica* in the natural watercress beds in central France. *Parasitology Research*, *87*(6), 475–478. <https://doi.org/10.1007/s004360100385>
295. Rosas-Hostos Infantes, L. R., Paredes Yataco, G. A., Ortiz-Martínez, Y., Mayer, T., Terashima, A., Franco-Paredes, C., Gonzalez-Diaz, E., Rodriguez-Morales, A. J., Bonilla-Aldana, D. K., Vargas Barahona, L., Grimshaw, A. A., Chastain, D. B., Sillau, S., Marcos, L. A., & Henao-Martínez, A. F. (2023). The global prevalence of human fascioliasis: a systematic review and meta-analysis. *Therapeutic Advances in Infectious Disease*, *10*. <https://doi.org/10.1177/20499361231185413>
296. Ross, J. G. (1970). The economics of Fasciola hepatica infections in cattle. *The British Veterinary Journal*, *126*(4), xiii–xixv.
297. Rubilar, L., Cabreira, A., & Giacaman, L. (1988). Treatment of *Fasciola hepatica* infection in horses with triclabendazole. *The Veterinary Record*, *123*(12), 320–321. <https://doi.org/10.1136/vr.123.12.320>
298. Rufino-Moya, P. J., Zafra Leva, R., Martínez-Moreno, Á., Buffoni, L., Valderas García, E., Pérez Arévalo, J., Molina-Hernández, V., Ruiz-Campillo, M. T., Herrera-Torres, G., & Martínez-Moreno, F. J. (2024). Advancement in Diagnosis, Treatment, and Vaccines against *Fasciola hepatica*: A Comprehensive Review. *Pathogens (Basel, Switzerland)*, *13*(8). <https://doi.org/10.3390/pathogens13080669>
299. Rushton, B. (1977). Ovine fascioliasis following reinfection. *Research in Veterinary Science*, *22*(1), 133–134.
300. Saberinasab, M., Mohebali, M., Molawi, G., Kia, E. B., Aryaeipour, M., & Rokni, M. B. (2014). Seroprevalence of Human Fascioliasis Using Indirect ELISA in Isfahan District, Central Iran In 2013. *Iranian Journal of Parasitology*, *9*(4).
301. Sabourin, E., Alda, P., Vázquez, A., Hurtrez-Boussès, S., & Vittecoq, M. (2018). Impact of Human Activities on Fasciolosis Transmission. *Trends in Parasitology*, *34*(10), 891–903. <https://doi.org/10.1016/j.pt.2018.08.004>
302. Sağmak Tartar, A., Aşan, M. A., Bozdağ, A., Bahçecioğlu, İ. H., Akbulut, A., & Demirdağ, K. (2025). *Fasciola hepatica* Diagnosis: Clinical and Laboratory Clues from A University Hospital Experience. *Turkiye Parazitolojii Dergisi*, *49*(2), 58–62. <https://doi.org/10.4274/tpd.galenos.2025.42650>
303. Şahin, M., Afshar, M. T., Yıldız, R., Aydemir, S., Yılmaz, H., & Taş Cengiz, Z. (2023). Prevalence of Fascioliasis in Slaughtered Cattle in Ağrı Province. *Turkiye Parazitolojii Dergisi*, *47*(3), 156–159. <https://doi.org/10.4274/tpd.galenos.2023.39306>
304. Saksonov, S. I., & Saksonova, E. E. (1985). [Difficulties in the diagnosis of fascioliasis]. *Klinicheskaia Meditsina*, *63*(7), 73–76.
305. Saleha, A. A. (1991). Liver fluke disease (fascioliasis): epidemiology, economic impact and public health significance. *The Southeast Asian Journal of Tropical Medicine and Public Health*, *22 Suppl*, 361–364.
306. Salvador, F., Serres, X., Goterris, L., Martínez-Campreciós, J., Espinosa-Pereiro, J., Esteban, E., & Molina, I. (2025). Acute fascioliasis in a returning traveller from Ethiopia. *Journal of Travel Medicine*, *32*(1). <https://doi.org/10.1093/jtm/taae132>
307. Samson, K. S., Wilson, G. I., & Allen, R. W. (1969). Sheep liver fluke anthelmintics: preliminary experiments with eight compounds against mature and immature *Fasciola hepatica*. *American Journal of Veterinary Research*, *30*(5), 807–810.
308. Sanei Taheri, M., & Davarpanah, A. H. (2020). Biliary fascioliasis. *Surgery*, *167*(5), e13–e14. <https://doi.org/10.1016/j.surg.2020.01.021>
309. Sanyal, P. K. (1995). Kinetic disposition of triclabendazole in buffalo compared to cattle. *Journal of Veterinary Pharmacology and Therapeutics*, *18*(5), 370–374. <https://doi.org/10.1111/j.1365-2885.1995.tb00606.x>
310. Sargison, N. (2012). Diagnosis of triclabendazole resistance in *Fasciola hepatica*. *The Veterinary Record*, *171*(6), 151–152. <https://doi.org/10.1136/vr.e5357>
311. Sarkari, B., & Khabisi, S. A. (2017). Immunodiagnosis of Human Fascioliasis: An Update of Concepts and Performances of the Serological Assays. *Journal of Clinical and Diagnostic Research : JCDR*, *11*(6), OE05–OE10. <https://doi.org/10.7860/JCDR/2017/26066.10086>
312. Savioli, L., Chitsulo, L., & Montresor, A. (1999). New opportunities for the control of fascioliasis. *Bulletin of the World Health Organization*, *77*(4), 300.
313. Schillhorn van Veen, T. W. (1980). Dynamics of Lymnaea natalensis populations in the Zaria area (Nigeria) and the relation to Fasciola gigantica infections. *Acta Tropica*, *37*(2), 183–194.
314. Schillhorn van Veen, T. W., & Buys, J. (1979). The serodiagnosis of chronic fascioliasis (*Fasciola gigantica*) using a fluorescent-antibody technique with single and multiple whole-fluke antigens. *Tropenmedizin Und Parasitologie*, *30*(2), 194–197.
315. Schweizer, G., Braun, U., Deplazes, P., & Torgerson, P. R. (2005). Estimating the financial losses due to bovine fasciolosis in Switzerland. *The Veterinary Record*, *157*(7), 188–193. <https://doi.org/10.1136/vr.157.7.188>
316. Selemetas, N., Phelan, P., O’Kiely, P., & Waal, T. d. (2014). Weather and soil type affect incidence of fasciolosis in dairy cow herds. *The Veterinary Record*, *175*(15), 371. <https://doi.org/10.1136/vr.102437>
317. Sergiev, V. P., Uspenskiĭ, A. V., Sorokina, N. P., Syskova, T. G., Gorokhov, V. V., Romanenko, N. A., & Molchanov, I. A. (2004). [Human fascioliasis: status of the problem]. *Meditsinskaia Parazitologiia I Parazitarnye Bolezni*, *3*, 52–58.
318. Setiawan, H., Kusnoto, & Hariadi, M. (2019). The Effect of Breeding Management on the Prevalence and Trematode infection Degree in calves. *Indian Journal of Public Health Research & Development*, *10*(9), 1535. <https://doi.org/10.5958/0976-5506.2019.02667.6>
319. Sewell, M. M. (1966). The pathogenesis o fascioliasis. *The Veterinary Record*, *78*(3), 98–105. <https://doi.org/10.1136/vr.78.3.98>
320. Sewell, M. M., & Hammond, J. A. (1972). The detection of *Fasciola* eggs in faeces. *The Veterinary Record*, *90*(18), 510–511. <https://doi.org/10.1136/vr.90.18.510>
321. Shehab, A. Y., Basha, L. M. A., El-Morshedy, H. N., Abdel-Fattah, M., Osman, M. M., & Farag, H. F. (1999). Circulating antibodies and antigens correlate with egg counts in human fascioliasis. *Tropical Medicine & International Health*, *4*(10), 691–694. <https://doi.org/10.1046/j.1365-3156.1999.00466.x>
322. Shi, H., Li, M., Huang, X., Yao, C., Chen, X., Du, A., & Yang, Y. (2020). Development of SYBR Green real-time PCR for diagnosis of fasciolosis in sheep. *Veterinary Parasitology*, *283*. <https://doi.org/10.1016/j.vetpar.2020.109193>
323. Siles-Lucas, M., Becerro-Recio, D., Serrat, J., & González-Miguel, J. (2021). Fascioliasis and fasciolopsiasis: Current knowledge and future trends. *Research in Veterinary Science*, *134*, 27–35. <https://doi.org/10.1016/j.rvsc.2020.10.011>
324. Sillat, M. S., & Govarthanan, S. (2026). An unusually large liver abscess: A rare presentation of fascioliasis. *Tropical Doctor*, *56*(1), 105–106. <https://doi.org/10.1177/00494755251374464>
325. Singh, D. K., Singh, V. K., Singh, R. N., & Kumar, P. (2021). *Fasciolosis : causes, challenges and controls*. Springer. <https://doi.org/10.1007/978-981-16-0259-7>
326. Singh, K., Singh, A., & Singh, D. K. (1996). Molluscicidal activity of neem ( *Azadirachta indica* A.Juss). *Journal of Ethnopharmacology*, *52*(1), 35–40. <https://doi.org/10.1016/0378-8741(96)01383-9>
327. Singh, N. V., Singh, A., & Singh, V. K. (2024). Laboratory Assessment of Molluscicidal Activities of *Cannabis sativa* , *Acacia nilotica* , and *Tinospora cordifolia* Against Snail Host of *Fasciola* spp. *Vector-Borne and Zoonotic Diseases*, *24*(6), 382–389. <https://doi.org/10.1089/vbz.2023.0073>
328. Singh, S. K., Yadav, R. P., & Singh, A. (2010). Molluscicides from some common medicinal plants of eastern Uttar Pradesh, India. *Journal of Applied Toxicology*, *30*(1), 1–7. <https://doi.org/10.1002/jat.1498>
329. Smeal, M. G., & Hall, C. A. (1983). The activity of triclabendazole against immature and adult Fasciola hepatica infections in sheep. *Australian Veterinary Journal*, *60*(11), 329–331. <https://doi.org/10.1111/j.1751-0813.1983.tb02833.x>
330. Smith, G. (1984). The relationship between the size of Ly*mnaea truncatula* naturally infected with *Fasciola hepatica* and the intensity and maturity of the redial infection. *Journal of Helminthology*, *58*(2), 123–127. <https://doi.org/10.1017/s0022149x00028625>
331. Soosaraei, M., Fakhar, M., Teshnizi, S. H., Emameh, R. Z., Hezarjaribi, H. Z., Asfaram, S., Faridnia, R., & Kalani, H. (2020). Status of fasciolosis among domestic ruminants in Iran based on abattoir data: a systematic review and meta-analysis. *Annals of Parasitology*, *66*(1), 77–86. https://doi.org/10.17420/ap6601.240
332. Stansfield, D. G., & Lowndes, P. A. (1986). Strategic summer dosing for liver fluke. *The Veterinary Record*, *118*(22), 620. <https://doi.org/10.1136/vr.118.22.620>
333. Starr, J. R. (1981). Weather and animal well-being. *The Veterinary Record*, *109*(3), 49–50. https://doi.org/10.1136/vr.109.3.49
334. Stuen, S., Ersdal, C., Lacasta, D., & Ortín, A. (2022). Fasciolosis—An Increasing Challenge in the Sheep Industry. *Animals : An Open Access Journal from MDPI*, *12*(12). <https://doi.org/10.3390/ani12121491>
335. Suhardono, Widjajanti, S., Stevenson, P., & Carmichael, I. H. (1991). Control of *Fasciola gigantica* with triclabendazole in Indonesian cattle. *Tropical Animal Health and Production*, *23*(4), 217–220. <https://doi.org/10.1007/BF02357103>
336. Sukhdeo, M. V. K., Sangster, N. C., & Mettrick, D. F. (1988). Permanent feeding sites of adult *Fasciola hepatica* in rabbits? *International Journal for Parasitology*, *18*(4), 509–512. <https://doi.org/10.1016/0020-7519(88)90015-X>
337. Sun, P., Wronski, T., Apio, A., & Edwards, L. (2020). A holistic model to assess risk factors of fasciolosis in Ankole cattle. *Veterinary Parasitology, Regional Studies and Reports*, *22*, 100488. <https://doi.org/10.1016/j.vprsr.2020.100488>
338. Sunita, K., & Singh, D. K. (2011). Fascioliasis control: in vivo and in vitro phytotherapy of vector snail to kill fasciola larva. *Journal of Parasitology Research*, *2011*, 240807. <https://doi.org/10.1155/2011/240807>
339. Swarup, D., Pachauri, S. P., Sharma, B., & Bandhopadhyay, S. K. (1987). Serodiagnosis of *Fasciola gigantica* infection in buffaloes. *Veterinary Parasitology*, *24*(1-2), 67–74. <https://doi.org/10.1016/0304-4017(87)90131-2>
340. Taira, N., Yoshifuji, H., & Boray, J. C. (1997). Zoonotic potential of infection with *Fasciola* spp. by consumption of freshly prepared raw liver containing immature flukes. *International Journal for Parasitology*, *27*(7), 775–779. <https://doi.org/10.1016/s0020-7519(97)00038-6>
341. Takang, E. E., LeBreton, M., Ayuk, C. E., & MacLeod, E. T. (2019). A socio-economic study of Fasciola infections in cattle and sheep at the Etoudi slaughterhouse, Yaoundé, Cameroon. *Journal of Helminthology*, *94*, e92. <https://doi.org/10.1017/S0022149X19000890>
342. Takeuchi-Storm, N., Denwood, M., Petersen, H. H., Enemark, H. L., Stensgaard, A.-S., Sengupta, M. E., Beesley, N. J., Hodgkinson, J., Williams, D., & Thamsborg, S. M. (2018). Patterns of *Fasciola hepatica* infection in Danish dairy cattle: implications for on-farm control of the parasite based on different diagnostic methods. *Parasites & Vectors*, *11*(1), 1–18. <https://doi.org/10.1186/s13071-018-3248-z>
343. Tanabe, M. B., Caravedo, M. A., Clinton White, A., & Cabada, M. M. (2024). An Update on the Pathogenesis of Fascioliasis: What Do We Know? *Research and Reports in Tropical Medicine*, *15*, 13–24. <https://doi.org/10.2147/RRTM.S397138>
344. Taylor, S. M., & Ross, J. G. (1970). A survey of the Lymnaea truncatula habitat areas in Co. Tyrone and Co. Fermanagh, Northern Ireland. *The British Veterinary Journal*, *126*(8), xxix–xxxi.
345. Teixeira, J. N., Barcellos, R. R., Link, D. T., Mathias, L. A., Tobias, F. L., Braga, F. R., & Rossi, G. A. M. (2023). Prevalence of bovine fascioliasis and economic losses in an abattoir located in the state of Espírito Santo, Brazil. *Ciência Rural*, *53*(4). <https://doi.org/10.1590/0103-8478cr20220121>
346. Torresi, J., Richards, M. J., Taggart, G. J., & Smallwood, R. A. (1996). *Fasciola hepatica* liver infection in a Victorian dairy farmer. *The Medical Journal of Australia*, *164*(8), 511. <https://doi.org/10.5694/j.1326-5377.1996.tb122150.x>
347. Tumusiime, M., Manishimwe, J. C., & Ntampaka, P. (2023). Prevalence of Bovine Trematodiases and Associated Risk Factors in Nyagatare District, Rwanda. *Veterinary Medicine : Research and Reports*, *14*, 221–231. <https://doi.org/10.2147/VMRR.S430581>
348. Vázquez, A. A., Alba, A., Alda, P., Vittecoq, M., & Hurtrez-Boussès, S. (2022). On the arrival of fasciolosis in the Americas. *Trends in Parasitology*, *38*(3), 195–204. <https://doi.org/10.1016/j.pt.2021.12.001>
349. Venturina, V. M., Alejandro, M. A. F., Baltazar, C. P., Abes, N. S., & Mingala, C. N. (2015). Evidence of *Fasciola* spp. resistance to albendazole, triclabendazole and bromofenofos in water buffaloes (*Bubalus bubalis*). *Annals of Parasitology*, *61*(4), 283–289. <https://doi.org/10.17420/ap6104.20>
350. Vignoles, P., Favennec, L., Dreyfuss, G., & Rondelaud, D. (2002). Highland populations of *Lymnaea truncatula* infected with *Fasciola hepatica* survive longer under experimental conditions than lowland ones. *Parasitology Research*, *88*(4), 386–388. <https://doi.org/10.1007/s00436-001-0542-y>
351. Villavicencio, A. F., Bargues, M. D., Artigas, P., Guamán, R., Ulloa, S. M., Romero, J., Osca, D., & Mas-Coma, S. (2019). Lymnaeid Snail Vectors of Fascioliasis, Including the First Finding of *Lymnaea neotropica* in Ecuador, Assessed by Ribosomal DNA Sequencing in the Southern Zone Close to the Peru Border. *Acta Parasitologica*, *64*(4), 839–849. <https://doi.org/10.2478/s11686-019-00104-1>
352. Walker, S. M., Johnston, C., Hoey, E. M., Fairweather, I., Borgsteede, F. H. M., Gaasenbeek, C. P. H., Prodohl, P. A., & Trudgett, A. (2011). Potential rôle of hares in the spread of liver fluke in the Netherlands. *Veterinary Parasitology*, *177*(1-2), 179–181. <https://doi.org/10.1016/j.vetpar.2010.11.043>
353. Walker, S. M., Makundi, A. E., Namuba, F. V., Kassuku, A. A., Keyyu, J., Hoey, E. M., Prödohl, P., Stothard, J. R., & Trudgett, A. (2008). The distribution of *Fasciola hepatica* and *Fasciola gigantica* within southern Tanzania - constraints associated with the intermediate host. *Parasitology*, *135*(4), 495–503. <https://doi.org/10.1017/S0031182007004076>
354. Weisenberg, S. A., & Perlada, D. E. (2013). Domestically acquired fascioliasis in northern California. *The American Journal of Tropical Medicine and Hygiene*, *89*(3), 588–591. <https://doi.org/10.4269/ajtmh.13-0069>
355. Wessely, K., Reischig, H. L., Heinerman, M., & Stempka, R. (1988). Human fascioliasis treated with triclabendazole (Fasinex) for the first time. *Transactions of the Royal Society of Tropical Medicine and Hygiene*, *82*(5), 743–744. <https://doi.org/10.1016/0035-9203(88)90222-2>
356. Whitelaw, A., & Fawcett, A. R. (1981). Further studies in the control of ovine fascioliasis by strategic dosing. *The Veterinary Record*, *109*(6), 118–119. <https://doi.org/10.1136/vr.109.6.118>
357. Windsor, R. S., & Scott, W. A. (1976). Fascioliasis and Salmonellosis in African Elephants in Captivity. *British Veterinary Journal*, *132*(3), 313–317. <https://doi.org/10.1016/S0007-1935(17)34691-2>
358. Wszelaki, S., Podobiński, P., & Środoń, K. (2023). Molluscicidal activity of plant alkaloids. *Journal of Applied Toxicology : JAT*, *43*(12), 1778–1792. <https://doi.org/10.1002/jat.4466>
359. Yao, J. W., & Jia, T. W. (2023). [Global distribution and transmission of Fasciola]. *Zhongguo Xue Xi Chong Bing Fang Zhi Za Zhi = Chinese Journal of Schistosomiasis Control*, *34*(6), 654–658. <https://doi.org/10.16250/j.32.1374.2022234>
360. Yilmaz, H., Oner, A. F., Akdeniz, H., & Arslan, S. (1998). The effect of triclabendazole (Fasinex) in children with fasciolosis. *Journal of the Egyptian Society of Parasitology*, *28*(2), 497–502.
361. Youssef, F. G., Mansour, N. S., & Aziz, A. G. (1991). Early diagnosis of human fascioliasis by the detection of copro-antigens using counterimmunoelectrophoresis. *Transactions of the Royal Society of Tropical Medicine and Hygiene*, *85*(3), 383–384. <https://doi.org/10.1016/0035-9203(91)90300-n>
362. Zaraei, M., Arefkhah, N., Moshfe, A., Ghorbani, F., Mikaeili, F., & Sarkari, B. (2019). Prevalence of bovine fascioliasis in a new-emerging focus of human fascioliasis in BoyerAhmad district, southwest of Iran. *Comparative Immunology, Microbiology and Infectious Diseases*, *66*. <https://doi.org/10.1016/j.cimid.2019.101350>
363. Zhang, K., Liu, N., Liu, S.-H., Gong, Y.-H., Hou, N., Sun, J.-J., & Wu, F. (2011). [Snail control effect of film covering method in fish ponds and ditches]. *Zhongguo Xue Xi Chong Bing Fang Zhi Za Zhi = Chinese Journal of Schistosomiasis Control*, *23*(6), 695–696, 707.
364. Zoghi, S., Emami, M., Shahriarirad, S., Vahedi, R., Cheraghi, M. R., Zamiri, B., Arefkhah, N., Ghorbani, F., & Sarkari, B. (2019). Human fascioliasis in nomads: A population-based serosurvey in southwest Iran. *Le Infezioni in Medicina*, *27*(1), 68–72.
